# Global characterization of the Dicer-like protein DrnB roles in miRNA biogenesis in the social amoeba *Dictyostelium discoideum*

**DOI:** 10.1080/15476286.2018.1481697

**Published:** 2018-08-21

**Authors:** Zhen Liao, Jonas Kjellin, Marc P. Hoeppner, Manfred Grabherr, Fredrik Söderbom

**Affiliations:** a Department of Cell and Molecular Biology, Uppsala University, Uppsala, Sweden; b Department of Medical Biochemistry and Microbiology, Uppsala University, Uppsala, Sweden; c Christian-Albrechts-University of Kiel, Institute of Clinical Molecular Biology, Kiel, Germany

**Keywords:** Dicer, microRNA, amoeba, biogenesis, evolution, transcriptional start site, intron, development, Dictyostelium discoideum

## Abstract

Micro (mi)RNAs regulate gene expression in many eukaryotic organisms where they control diverse biological processes. Their biogenesis, from primary transcripts to mature miRNAs, have been extensively characterized in animals and plants, showing distinct differences between these phylogenetically distant groups of organisms. However, comparably little is known about miRNA biogenesis in organisms whose evolutionary position is placed in between plants and animals and/or in unicellular organisms. Here, we investigate miRNA maturation in the unicellular amoeba *Dictyostelium discoideum*, belonging to Amoebozoa, which branched out after plants but before animals. High-throughput sequencing of small RNAs and poly(A)-selected RNAs demonstrated that the Dicer-like protein DrnB is required, and essentially specific, for global miRNA maturation in *D. discoideum*. Our RNA-seq data also showed that longer miRNA transcripts, generally preceded by a T-rich putative promoter motif, accumulate in a *drnB* knock-out strain. For two model miRNAs we defined the transcriptional start sites (TSSs) of primary (pri)-miRNAs and showed that they carry the RNA polymerase II specific m^7^G-cap. The generation of the 3ʹ-ends of these pri-miRNAs differs, with pri-mir-1177 reading into the downstream gene, and pri-mir-1176 displaying a distinct end. This 3´-end is processed to shorter intermediates, stabilized in DrnB-depleted cells, of which some carry a short oligo(A)-tail. Furthermore, we identified 10 new miRNAs, all DrnB dependent and developmentally regulated. Thus, the miRNA machinery in *D. discoideum* shares features with both plants and animals, which is in agreement with its evolutionary position and perhaps also an adaptation to its complex lifestyle: unicellular growth and multicellular development.

## 1. Introduction

MicroRNAs (miRNAs) are ~ 21 nucleotides (nts) long RNAs, which have been mostly studied in animals and plants where they act as crucial regulators of gene expression. Their importance is underscored by the estimation that more than half of the human genes are regulated by miRNAs [1]. These small regulatory RNAs guide Argonaute effector proteins to target mRNAs via base-pairing, which in turn leads to destabilization of the target RNA and/or translational inhibition [2,3]. In both plants and animals, most miRNAs derive from stem-loop structures embedded in primary RNA (pri-RNA) transcribed by RNA polymerase II (Pol II) [4,5].

Although there are many similarities between miRNA biogenesis and function in plants and animals, there are also distinct differences [6]. In plants, miRNA loci are most often intergenic while in animals, miRNA commonly derive from introns of protein coding genes or long noncoding (lnc)RNAs [7–9]. Processing of miRNAs also differs. In animals, the stem-loop structure residing in the pri-miRNA is recognized by the microprocessor, a complex consisting of the RNase III enzyme Drosha and an RNA-binding protein [10–13]. Drosha endonucleolytically cleaves out the stem-loop structure, the precursor-miRNA (pre-miRNA), which is exported out of the nucleus by Exportin-5 [14]. In the cytoplasm, another RNase III-like enzyme, Dicer, processes the pre-miRNA to mature miRNAs [15,16]. In contrast, in plants both cleavage steps take place in the nucleus [17,18]. The stem-loop, harboring the miRNA sequence, is recognized by a complex of proteins where Dicer-like protein DCL1 first releases the pre-miRNA and subsequently also cleaves the pre-miRNA to generate miRNAs. These miRNAs are transferred to the cytoplasm by HASTY, a homologue of Exportin-5 [18,19]. However, a revised model has recently been put forward where miRNAs are loaded onto AGO1 whereafter the complex is transported out of the nucleus by a HASTY-independent pathway [20].

Besides plants and animals, evidence for miRNAs in unicellular eukaryotes were first reported in the alga *Chlamydomonas reinhardtii* and by us in the unicellular social amoeba *Dictyostelium discoideum* [21–23]. *D. discoideum* has a fascinating life-cycle where growing single cells, upon starvation, are reprogrammed to enter multicellular development where up to 100 000 cells aggregate, differentiate, and finally form a fruiting body consisting of a stalk topped by a ball of spores [24]. Phylogenetically, *D. discoideum* belongs to the supergroup Amoebozoa, which branched out after plants but before fungi and animals [25,26].

It is commonly thought that miRNAs evolved by convergent evolution, however recent reports suggest that miRNAs existed already in the last common ancestor of plants and animals [27]. Studies of miRNAs and their biogenesis in lineages other than animals and plants and in organisms that hover between uni- and multicellularity should give further insights into evolution and function of miRNAs. Given the differences in miRNA maturation between plants and animals, the intermediate position of *D. discoideum* in the evolution of eukaryotes as well as its ability to transit from unicellular to multicellularity adds great interest to investigate how miRNAs are generated in this organism. Furthermore, very little is known about miRNA biogenesis in unicellular organisms.

Although increasing numbers of *D. discoideum* miRNAs have been reported by us and others during the past decade [22,28,29], miRNA biogenesis is still incompletely characterized in this organism. We previously reported that one of the two *D. discoideum* Dicer-like proteins, DrnB, is required for maturation of a few miRNAs tested [22,28]. DrnB has been shown to localize to the nucleus where it interacts with RbdB, a double stranded (ds) RNA binding domain containing protein [30]. Interestingly, the domain composition of DrnB is more similar to Drosha than e.g. Dicer-like proteins in plant, a feature shared with the DCL-3 involved in miRNA maturation in *C. reinhadtii* [30,31]. RbdB together with DrnB has been proposed to constitute the microprocessor in *D. discoideum* and depletion of RbdB abolishes the maturation of some tested miRNAs, similar to the results shown for strains where *drnB* has been disrupted [28,29]. However, evidence for the generality of DrnB in small RNA processing, such as miRNA maturation, has been lacking.

In this study, we have investigated the global DrnB-dependent biogenesis of all known miRNAs in *D. discoideum* as well as carried out a more detailed mechanistic study of two model miRNAs. For this we utilized high-throughput sequencing of small RNAs and poly(A)-selected RNA from wt and DrnB depleted (*drnB*^-^) cells both during unicellular growth and multicellular development. The observed overall depletion of miRNAs and the associated stabilization of pri-miRNA in *drnB*^-^ cells, allowed us to identify the start as well as termination of transcription of specific pri-miRNAs in *D. discoideum*. Furthermore, we discovered oligo(A)-tailed processing intermediates which accumulate in the absence of DrnB. Finally, several new miRNAs were identified of which each of the two intronic miRNA loci produce two sets of miRNAs, a set up resembling miRNA-offset RNAs (moRs) found in animals [32,33]. Collectively, our data give new insights into miRNA biogenesis in an unicellular organism, demonstrating that miRNA production is characterized by features found in plants as well as animals, emphasizing the evolutionary position of *D. discoideum*.

## 2. Results

### 2.1. Dicer-like protein DrnB is dispensable for production of most small RNA classes

**Figure 1.**
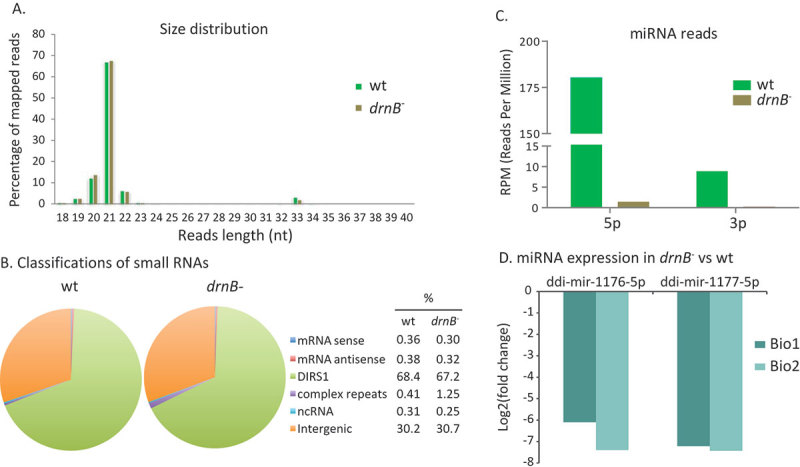
DrnB is required for miRNA biogenesis. (A) Size distribution of small RNA libraries from wt and *drnB*^-^ strains. For each strain, sequencing reads from biological replicates from growing (0h) and developed cells (16h) were pooled. (B) Classification of 21 nt reads where % depicts fraction of reads derived from classes of RNA or genomic locations compared to total number of 21 nt read. (C) Quantification of small RNA reads matching the mature mir-5p and 3p of our previously published miRNA [28]. (D) Expression of ddi-mir-1176-5p and ddi-mir-1177-5p in wt and *drnB*^-^ strains during growth (0h) quantified by stem-loop RT-qPCR (2 biological replicates). Each bar represents log2(fold change) (*drnB*^-^ vs wt).

We previously reported that the Dicer-like protein DrnB is required for maturation of four miRNAs in *D. discoideum* as assessed by northern blot analysis [22,28,29]. However, whether DrnB is essential for biogenesis of all miRNAs as well as for other small RNAs has not been analyzed. To address these questions, we performed high-throughput sequencing of small RNAs from wt (AX2) and DrnB depleted cells (the *drnB* knock-out construct is depicted in figure S1). To increase our chances of identifying also small RNAs only expressed at specific life stages, we performed small RNA-seq (biological duplicates) on total RNA from growing cells (0h) and slug/finger structures (16h) – a multicellular developmental stage. The size distribution showed distinct peaks at 21 nt of near equal proportion of reads from both wt and *drnB*^-^ strains (Figure 1(a)). No large difference was observed for 21 nt sequences matching mRNA (sense and antisense) or non-coding RNAs (ncRNAs) such as tRNAs, rRNAs, small nucleolar RNAs (snoRNAs), spliceosomal RNAs (snRNAs), single recognition particle RNAs (SRP RNAs) or Class I RNAs (Figure 1(b)) [22,26,34–36]. The great majority of the 21 nt RNAs from both wt and *drnB*^-^ strains (68,4% and 67,2%, respectively) derive from the retrotransposon element DIRS-1, which also may function as centromeres in *D. discoideum* [37,38]. This result corroborates our previous finding of large numbers of DIRS-1 small RNAs in *D. discoideum* wt cells [22,28,39,40]. Furthermore, about equal numbers of 21 nt RNAs from both strains (~ 30%) have their origin in intergenic regions, where genes for most of the previously identified miRNAs resides (see below) [22,28]. However, a notable difference between the wt and *drnB*^-^ strains is seen for complex repeats other than DIRS-1. In the *drnB*^-^ strain, complex repeat small RNAs constitute 1.25% of the total 21 nt RNAs as compared to 0.41% in wt cells (Figure 1(b)). This difference is mostly contributed by an increased population of small RNAs from the non-long terminal repeat (LTR) retrotransposon TRE3-A [41] in DrnB depleted cells (data not shown). Taken together, these results show that DrnB has no major overall effect on the small RNA population (besides miRNAs, see below) in *D. discoideum*, neither considering number of reads nor their origins.

### 2.2. DrnB is required for global maturation of previously identified miRNAs in D. discoideum

In an earlier study performed on wt cells we showed that miRNAs only made up a small fraction of the total RNA reads generated by small RNA-seq [22,28]. Consequently, a difference in the miRNA population between wt and the *drnB*^-^ strains may be concealed by the large number of DIRS-1 and other 21 nt sequences (Figure 1(a,b)). Thus, in order to understand if DrnB is required for biogenesis of all miRNAs in *D. discoideum*, we dissected the small RNA reads to investigate the miRNA populations alone. Corroborating the crucial role for DrnB in producing a few tested miRNAs, all of our 17 previously identified miRNAs [28] were dramatically reduced in the *drnB*^-^ strain. This was observed for both mir-5p and mir-3p derived from the 5´ and 3´ arms of the predicted pre-miRNA hairpins, respectively (Figure 1(c)). The reduction of the mir-5p in *drnB*^-^ cells for two miRNAs, ddi-mir-1176 and ddi-mir-1177 (from here on referred to as mir-1176 and mir-1177), used as model miRNAs throughout this study, was confirmed by stem-loop reverse transcription quantitative PCR (stem-loop RT-qPCR) (Figure 1(d)) [42]. In summary, our results strongly suggest that the Dicer-like protein DrnB is responsible for global miRNA generation in *D. discoideum*.

### 2.3. Identification of new DrnB dependent miRNAs

Based on our results demonstrating that the presence of DrnB is a general requirement for miRNA maturation in *D. discoideum*, we added this as one of the search criteria for discovery and verification of new miRNAs. First, all small RNA-seq data from wt cells were combined and subjected to different search strategies to identify miRNA candidates. For this, programs designed for detection of small/miRNA in animals and plants were used, i.e. miRDeep2 and ShortStack [43,44]. In addition, the small RNA-seq data was also analyzed utilizing our pipeline designed for *D. discoideum* miRNA discovery [28,45]. The resulting candidates were manually curated and filtered based on the stringent criteria defining high confidence miRNA specified by Kozomora and Griffith-Jones [46]. In brief: i) at least 10 reads mapping to mir-3p and mir-5p, ii) the most abundant reads must pair with a 0–4 nt 3´ overhang (however we used 2 nt 3ʹ overhang as a more strict criteria), iii) a common 5ʹ-end for at least 50% of the reads mapping to each arm of the hairpin, iv) minimum folding free energy of <-0.2 kcal/mol/nt for the hairpin precursor and, v) at least 60% of the double stranded miRNA must be paired in the hairpin precursor. Finally, miRNA candidates should be clearly downregulated in *drnB*^-^ cells. In total, 10 new miRNAs from eight miRNA loci were identified (some loci produce two miRNAs as described below). The miRNAs are mostly derived from intergenic loci but also, in a few cases, from introns (Table S1). With a few exceptions (see below) the mir-5p and the corresponding mir-3p fragments, matching the predicted pre-miRNA hairpin structures, fulfill all criteria of true miRNAs (Fig. S2 and Table S1). Furthermore, all mir-5p and mir-3p RNAs are down regulated in the *drnB*^-^ strain (Fig. S2). The reads from *drnB*^-^ cells often derive from many different parts of the predicted hairpin structures. Moreover, the 5´-ends of the mir-5p and mir-3p in the *drnB*^-^ strain are not well defined compared to the RNAs from the wt strain (Fig. S2).

Interestingly, the new miRNAs are developmentally regulated (Fig. S2), which was also shown for the previously identified miRNAs [28]. Furthermore, in the small RNA-seq libraries we also detected reads for the four miRNA candidates recently reported to be present in growing *D. discoideum* cells [29]. Two of these, miRNA_can_D1 and D2, fulfilled the high confidence miRNA criteria stated above and are differently expressed during development (data not shown).

Three of the new miRNA candidates failed to meet the criteria that at least 10 reads should map to the mir-5p and mir-3p. For mir-1178, mir-1180-1, and mir-1181 we identified 9, 9, and 2 reads, respectively, matching mir-3p. However, when we add the sequences from our previously reported SOLiD small RNA-seq libraries [28], both mir-1180-1 and mir-1181 fulfill all the criteria. We chose to include also mir-1178 as a new miRNA due to the wealth of siRNA from e.g. the transposon DIRS-1 (Figure 1(b)), which occludes the isolation of other less abundant small RNAs. Furthermore, all candidate miRNAs (including the three discussed above) are developmentally regulated and DrnB dependent (Table S1 and Fig. S2).

Interestingly, for the two intronic miRNAs, mir-1180 and the previously reported mir-7102 [28], we identified two mir-5p and mir-3p pairs matching each predicted hairpin structure (Fig. S2). These pairs are situated immediately adjacent to each other and are DrnB dependent, indicating that the same processing machinery is involved. This is similar to the extra single or pair of reads from pre-miRNAs, termed miRNA-offset RNAs (moRs), reported in the simple chordate *Ciona intestinalis* and later also in human and mouse [32,33,47]. For each pair, we named these mir-1180-1 and −2 and mir-7102–1 and −2 where ‘1’ designates the miRNA pair closest to the base of the predicted stem-loop. It should be noted that also mir-1185 shows a similar pattern where two pairs of mir-5p and mir-3p are produced from the same stem-loop, however the cleavage pattern is not as clear as for the mir-1180 and mir-7102 pairs and we decided to only include the most abundant pair as the miRNA duplex (Fig. S2). We reason that the imprecise processing of presumably two sets of miRNAs is the reason why the mir-1185-5p does not meet the criteria that at least 50% should have a common 5ʹ-end (Table S1).

In a recent report, Meier *et al*., demonstrated upregulation of miRNAs in *D. discoideum* cells where the gene for one of the Argonautes, AgnA, had been knocked out. The reason for this is unknown. Furthermore, when cells were depleted of the microprocessor component RbdB, miRNA levels were reduced as compared to wt cells [29]. We analyzed the RNA-seq data from the knock-out strains, deposited in NCBI Gene Expression Omnibus (GEO) Database (GSE56111), in order to investigate if our newly identified miRNAs are regulated in the same manner. The result indicates that seven of the ten new miRNAs reported here are more abundant in *agnA*^-^ cells and reduced in a *rbdB*^-^ strain as compared to wt cells (data not shown). Three of the miRNAs were not detected in the available RNA-seq data from the knock out strains, which is most likely due to the overall relatively low number of ~ 21 nt reads and the fact that these data are from growing cells while our analysis (see above) show that most miRNAs are developmentally upregulated.

**Figure 2.**
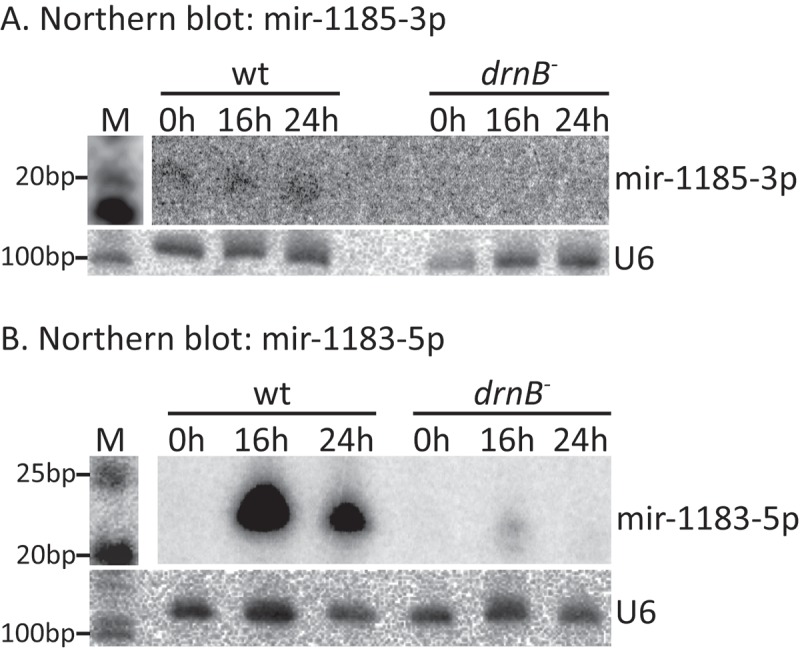
Expression of new miRNAs in wt and *drnB*^-^ strains during growth and development. (A,B) Northern blot analyses showing expression of mir-1185-3p (A) and mir-1183-5p (B) in wt and *drnB*^-^ strains during growth (0 h) and two developmental stages (16 h and 24 h). U6 spliceosomal RNA was probed and used as loading control.

The expression of two of the miRNAs, mir-1185-3p and mir-1183-5p, was verified by northern blot analysis. In order to simultaneously investigate developmental regulation, RNA from three different time points was extracted: 0h, 16h, and 24h fully developed fruiting bodies (Figure 2). As expected, both mir-1185-3p and mir-1183-5p were detected in wt cells but not (or very little) in *drnB*^-^ cells. Furthermore, mir-1183-5p was dramatically upregulated between 0h and 16h, corroborating the small RNA-seq data (Fig. S2). Taken together, 10 new miRNA candidates were identified of which 8 met all the high confidence criteria for true miRNAs. In addition, all miRNAs were DrnB dependent and developmentally regulated. Furthermore, two of the predicted miRNA hairpin structures generated each two sets of predicted miRNA duplexes situated directly adjacent to each other.

### 2.4. Transcripts from miRNA genes are stabilized in the absence of DrnB

To date, neither primary (pri-miRNA) nor precursor (pre-miRNA) have been experimentally defined in *D. discoideum* or, to our knowledge, in any other unicellular organism. Since *D. discoideum* apparently lacks a bona fide Drosha, required for generation of pre-miRNA in animals, the miRNA biogenesis may be more similar to that in plants where one Dicer-like enzyme is responsible for producing both the pre- and mature miRNAs [19,29,30,48]. Hence, we hypothesized that the reduction of miRNAs observed in DrnB depleted cells would be reflected in stabilization and accumulation of longer pri-miRNA transcripts not processed to mature miRNAs, allowing for subsequent analysis of their 5´- and 3´-ends. To this end, we performed high-throughput sequencing of poly(A) selected RNA from the same total RNA samples used for small RNA sequencing. Poly(A)-selection was employed to reduce rRNA contamination. We speculated that pri-miRNA transcripts would be present in our poly(A) RNA-selected libraries for two reasons. First, a recent report by Kruse *et al.* indicated that mir-1176 and mir-1177 are dependent on RNA polymerase II (RNA Pol II) transcription [30], suggesting that pri-miRNA transcripts in *D. discoideum* may be polyadenylated as has been demonstrated in animals and plants [4,5,49]. Secondly, the genome of *D. discoideum* is very AT-rich (about 78%) [26], where long stretches of As are common within transcripts and therefore most likely allow for selection of RNAs with internal poly(A)-tracts.

**Figure 3.**
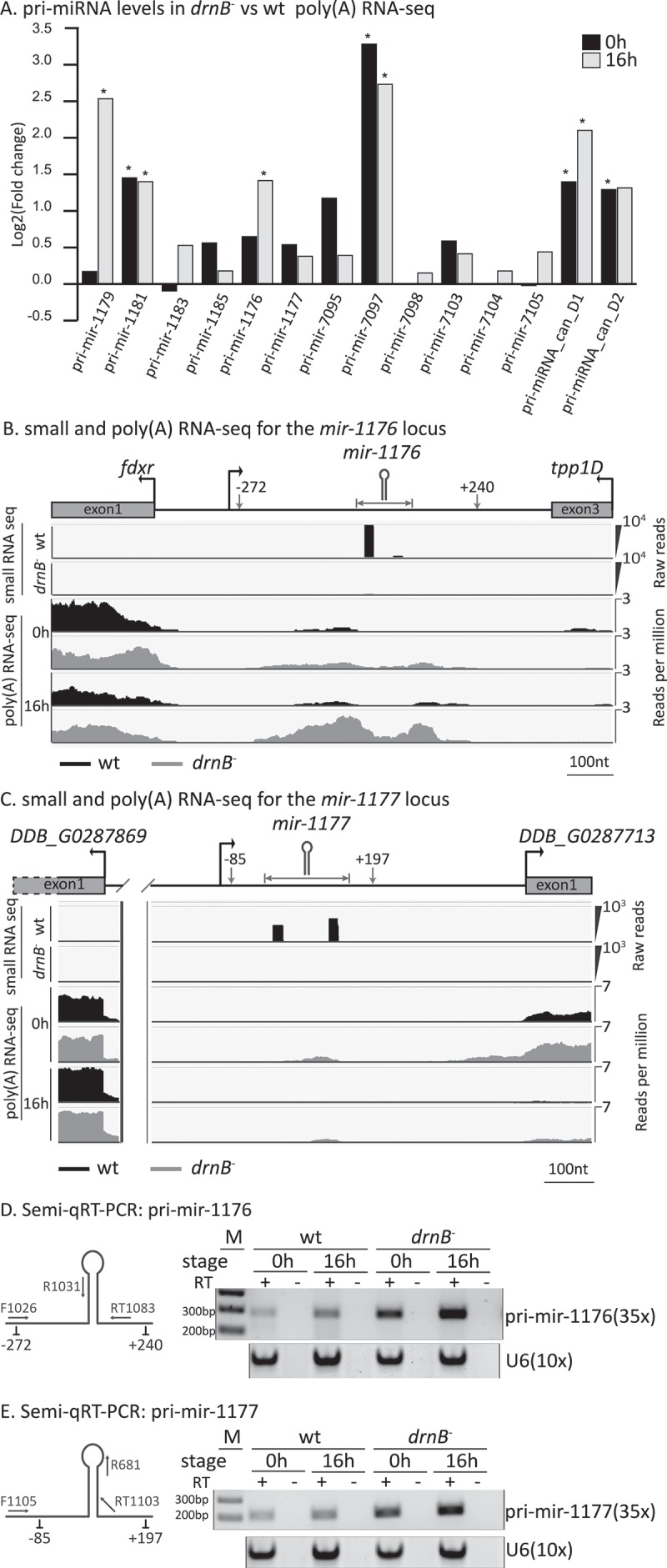
Transcripts from miRNA loci are enriched in the *drnB*^-^ strain. (A) Differential expression analysis of predicted miRNA precursor transcripts based on poly(A) RNA-seq data. Positive values indicate that the transcripts are enriched in DrnB depleted cells. * indicates false discovery rate (FDR) < 0.05. (B,C) Small RNA and poly(A) RNA-seq profiles across the mir-1176 and the mir-1177 loci. The miRNA loci and the beginning and/or end of the upstream and downstream annotated protein coding genes are indicated. Numbers specify start and end of reads covering the miRNA loci in relation to the 5´ most nucleotide of the mir-5p. The predicted miRNA hairpins are indicated by schematic structures and horizontal arrows. Small RNA-seq: for each strain, reads from two biological replicates from growing and developing cells, respectively, were pooled and are displayed as total read counts. Poly(A) RNA-seq: reads per million for pooled biological replicates from growing (0h) and developing (16h) cells, respectively, from wt (black) and *drnB*^-^ (grey). (D,E) Semi-qRT-PCR analysis of transcripts from miRNA genes. Left: schematic pictures of the predicted miRNA hairpins with adjacent regions; primers for reverse transcription (RT1083 and RT1103) and PCR primers marked F (forward) and R (reverse) are indicated; numbers below the schematic structure refer to start and end of poly(A) RNA-seq reads as described above. Right: Semi-qRT-PCR of transcripts from the mir-1176 and mir-1177 genes in wt and DrnB depleted (*drnB*^-^) cells from growing (0h) and 16h developed cells, respectively, analyzed by agarose gel electrophoresis. +/- RT indicates if reverse transcriptase was present or absent during the cDNA reactions. U6 spliceosomal RNA was used as reference. Numbers of PCR cycles are indicated.

Of the 27 miRNA loci identified so far in *D. discoideum* (see above), we chose to analyze the 19 loci that do not overlap with any other gene, e.g. that do not derive from introns or thug-S repetitive elements [28,29]. Indeed, in our poly(A)-selected RNA libraries we found reads extending across the predicted miRNA hairpin structure from 14 of these intergenic miRNA loci (for unknown reasons no reads were detected for the other intergenic loci). In addition, differential expression analysis showed upregulation of longer transcripts from the majority of the predicted miRNA loci in the absence of DrnB in both growing and developed cells (Figure 3(a)). This is exemplified in Figure 3(b,c) showing reads mapping to mir-1176 and mir-1177 genes and that these accumulate in *drnB*^-^ cells. These reads extend both upstream and downstream of the predicted miRNA-hairpin, indicative of the presence and, at least to some extent, length of the pri-miRNAs. It should be noted that the number of reads mapping to some of the miRNA genes is low, making those particular changes statistically uncertain. We confirmed the RNA-seq data for mir-1176 and mir-1177 genes by semi-quantitative RT-PCR (semi-qRT-PCR), adjusting the number of cycles to avoid saturation (Figure 3(d,e), and S3). The assay showed that transcripts from the miRNA genes indeed are stabilized in *drnB*^-^ cells, both during growth (0h) and development (16h). Furthermore, both pri-mir-1176 and 1177 are upregulated during wt development, which is mirrored by the previously reported developmental increase of mir-1176 and 1177 (Figure 3(d,e)) [22,28]. Our results are in line with a recent report where semi-qRT-PCR showed accumulation of transcripts from parts of the mir-1176 and 1177 genes in the absence of DrnB [29]. Taken together, miRNA transcripts not processed to mature miRNAs accumulate in strains depleted of DrnB, emphasizing the importance of DrnB for miRNA maturation in *D. discoideum*.

### 2.5. Start, end, and processing intermediates of mir-1176 pri-miRNA

Since our data show that in DrnB depleted cells longer transcripts harboring the miRNA stem-loops are stabilized and mature miRNAs are basically abolished, we reasoned that this would simplify the elucidation of miRNA biogenesis pathway(s) in *D. discoideum*. We started out by analyzing the pri-miRNA of one of the model miRNA, mir-1176.

#### 2.5.1. Transcriptional start site of pri-miRNA-1176

**Figure 4.**
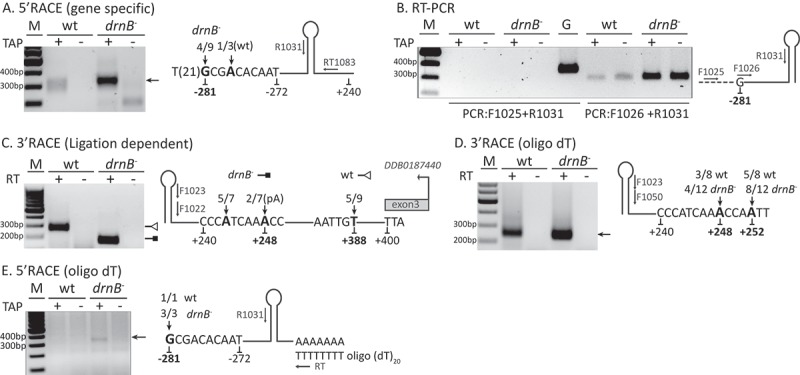
Pri-mir-1176 biogenesis in wt and *drnB*^-^ strains. Arrows in the schematic pri-miRNA-1176 figures represent primers used for the analyses where prefixes RT, F, and R stand for reverse transcription, forward, and reverse, respectively. Numbers below the sequences denote nucleotide positions in relation to the 5´ most nucleotide of mir-5p. Bold nucleotides and numbers indicate the determined transcriptional start site (TSS) and 3ʹ-ends. Additional numbering in regular font represent start and end of poly(A) RNA-seq reads covering the miRNA locus, except for +400 in C representing the last nucleotide of the stop codon for the downstream gene. 5ʹ and 3ʹ nucleotides from multiple RACE clones are indicated by numbers as fraction/total sequenced RACE-clones. Tobacco Acid Pyrophosphates (TAP) was used to remove cap-structures (A,B,E). (A) 5´RACE to determine the 5´-ends and TSS. (B) RT-PCR to confirm the presence of transcripts starting from the TSS. Genomic DNA (G) template was used as positive control. (C) 3´RACE to determine the 3´-ends. A DNA oligonucleotide was ligated to the 3´-ends of the RNA before reverse transcription followed by nested PCR. (D) 3ʹRACE to determine A-tailed sequences. An oligo (dT) primer was used for reverse transcription followed by nested PCR. (E) 5ʹRACE to determine 5´-ends of A-tailed pri-mir-1176 processing intermediates.

In the poly(A)-selected libraries from *drnB*^-^ cells, the 5´ most nucleotide covered by reads from the *mir-1176* gene is a T residue 272 nt from the start of mir-5p (Figure 3(b)). However, RNA Pol II transcripts often carry a 5ʹ-cap structure that would prevent the identification of the transcriptional start site (TSS) by our high-throughput sequencing approach. In order to pinpoint the very 5´-end, i.e the TSS, of the pri-miRNA-1176, we performed rapid amplification of cDNA ends (5´RACE). First, RNA was treated with calf intestinal alkaline phosphatase (CIP) to remove 5´ phosphate groups whereafter the RNA was treated with tobacco acid pyrophosphates (TAP) to remove cap structures and enable ligation of the RNA adapter oligo to the TSS. This was followed by RT-PCR, cloning, and Sanger sequencing. Samples not subjected to TAP treatment were used as controls. As expected, when transcripts from the mir-1176 gene were analyzed, more 5´RACE products were detected from the *drnB*^-^ strain as compared to the wt strain, supporting an enrichment of pri-miRNAs in DrnB depleted cells (Figure 4(a)). Importantly, none of the longer PCR-products were detected from samples not treated with TAP, indicating that the transcripts carry a 5´-cap. The cloned 5ʹRACE sequences show a few different 5´ ends indicating more than one TSSs, which is common for Pol II transcripts in mammals and has been shown for miRNA genes in Arabidopsis [5,50]. Here, we inferred the most abundant and the 5´ most nucleotide as the start of transcription, hence the TSS of pri-mir-1176, begins with a G-residue, 281 nt from the 5´-end of mir-1176-5p (Figure 4(a)). Situated upstream of the TSS is a long run of 21 consecutive Ts, which may constitute a promoter motif of miRNA genes in *D. discoideum* (see below). It should be noted that under these experimental conditions, the identified start site was not detected among the very few 5´RACE products from wt cells. In this case the 5´-most RACE product was located 278 nt from the start of mir-1176-5p and may indicate TSS of pri-miRNAs which are less efficiently processed by DrnB (Figure 4(a) and S4A). To further confirm the start of the transcription, we analyzed if any transcripts upstream of the TSS could be detected. For this we used the cDNA generated for the 5´RACE reactions described in Figure 4(a) but different forward primers, placed upstream and downstream of the predicted TSS. As expected, amplification products could only be detected when the forward primer was positioned after the predicted TSS (Figure 4(b)). Furthermore, the PCR products were more abundant when cDNA template from *drnB*^-^ cells was used again supporting stabilization the pri-miRNA in the absence of DrnB. Taken together, these results strongly suggest that the transcription of pri-mir-1176 starts from a G residue situated 281 nt from the mature mir-1176-5p and that the TSS is preceded by a stretch of Ts.

#### 2.5.2. 3´-end of pri-mir-1176 and oligoadenylated processing intermediates

Having established the transcriptional start site of pri-mir-1176, we turned our attention to its 3´-end. Here, we used 3´RACE to analyze RNA from wt and *drnB*^-^ strains, where a DNA oligonucleotide was ligated to the 3´-end of the RNA, followed by reverse transcription and PCR amplification. As expected, the 3´RACE products from *drnB*^-^ cells seemed more abundant but notably, also shorter compared to those from wt cells (Figure 4(c)). Sequencing of the RACE-products revealed several distinct 3´-ends. In wt cells, the longest transcript represented by five out of nine clones, ended 388 nt from the 5ʹ-end of mir-1176-5p, which was also 12 nt upstream of the predicted stop codon of the downstream gene situated on the opposite DNA strand (Figure 4(c)). The remaining four mapped ends were scattered further upstream, between 341 and 373 nucleotides downstream of the start of mir-1176-5p (Fig. S4B). As expected from the gel electrophoresis analysis of the 3´RACE products, the transcripts from the *drnB*^-^ cells ended even closer to the mir-5p, at position 243 and 248 (five and two out of seven clones, respectively) (Figure 4(c)). These 3´-ends most likely represent processing intermediates generated by unknown ribonuclease(s). Interestingly, the two 3´RACE clones ending at position 248 in *drnB*^-^ cells were extended by short oligo(A) tails, 4 to 6 residues long. It should be noted that this region harbors three templated consecutive A residues and hence the actual 3´-end and the exact number of post-transcriptionally added adenosins could not be determined. For simplicity, the last A-residue at position 248 is indicated as the templated 3´-end of these transcripts (Figure 4(c)).

The 3´RACE results for *drnB*^-^ cells were fairly consistent with the poly(A) selected RNA-seq analysis where reads from the RNA libraries extended to 240 nt downstream of the first nt of mir-1176-5p (Figure 3(b)). However, there was a discrepancy between the RNA-seq results and the 3´RACE experiment for wt cells, showing that the 3ʹ-ends of the longest transcripts were positioned around nucleotide 240 and 388, respectively, downstream of the miRNA. This difference could reflect a very rapid processing of the 3´-end of the pri-miRNA, and thus that the longer transcripts were not picked up by the RNA-sequencing due to limited sequencing depth and/or it may be due to absence or presence of a poly(A)-tail on pri-mir-1176. In order to analyze this, we performed a modified 3ʹRACE to exclusively identify A-tailed RNAs. Instead of ligating an oligonucleotide to the 3´-end, we used an oligo(dT) primer for reverse transcription, designed to allow for sequencing of the 3´-ends of A-tailed transcripts. In contrast to the ligation dependent 3´RACE experiment (Figure 4(c)), no major differences in length of the oligo(dT) selected 3ʹRACE products from wt and *drnB*^-^ strains could be observed (Figure 4(d)). Interestingly, 3´-ends with A-tails were present not only in *drnB*^-^cells but also in wt cells and the A-tailed ends from both strains were confined to a narrow interval of five nucleotides (Figure 4(d)). Furthermore, in addition to the oligoadenylated nucleotide(s) identified by ligation dependent 3´RACE in *drnB*^-^ cells, oligo(dT) selected RNA revealed a second A-tailed site four to five nucleotides downstream (Figure 4(d)). Taken together, these results suggest that pri-mir-1176 is transcribed as a longer non-polyadenylated transcript. Shorter 3´-ends are stabilized in the absence of DrnB and these fragments can be A-tailed, in both wt and *drnB*^-^ cells.

#### 2.5.3. A-tailed processing intermediates have full-length capped 5´-ends

The presence of post-transcriptionally added A-tails to 3ʹ-ends of mir-1176 processing intermediates prompted us to analyze the very 5ʹ-end of these RNAs. This would reveal if these A-tailed precursors carry a 5ʹ-cap structure from the pri-miRNA or if they have been processed also at the 5ʹ-end. In order to answer this we used a slightly different 5ʹRACE approach to capture the 5´-ends of the A-tailed processing intermediates. After TAP-treatment and ligation of the RNA oligonucleotide to the 5´-ends, we used an oligo(dT) primer (instead of a gene specific primer) to select for A-tailed RNA for subsequent cDNA conversion. The cDNA products generated from transcripts from the mir-1176 gene were amplified using a reverse primer placed in the 5´ part of the stem-loop. The method allowed for identification not only of capped RNA but also cleavage intermediates carrying 5ʹ-monophosphates, like canonical pre-miRNAs in animals. Surprisingly, 5ʹRACE products were only present in TAP-treated samples from *drnB*^-^ cells, indicating that these A-tailed transcripts have a 5ʹ-cap structure (Figure 4(e)). Sequencing of the cloned 5ʹRACE products revealed that all transcripts started with the G at position −281, which we defined as the first nucleotide of pri-mir-1176 (see above). Even though RNA isolated from wt cells did not give rise to any apparent RACE result, putative PCR products of the same size from wt strain was also extracted from the gel and sequenced. Only one sequence could be retrieved and this started with the same G residue at position −281 (Figure 4(e)). Taken together, the results suggest that pri-mir-1176 is transcribed as 669 nt long 5´ capped pri-miRNA. The pri-miRNA is subsequently processed downstream of the hairpin by one or several unknown RNase(s) and these processing intermediates can be oligoadenylated. Furthermore, the A-tailed processing intermediates are capped and may constitute pri-miRNAs or possibly aberrant RNA marked for destruction (see Discussion).

### 2.6. Start and end of pri-mir-1177

In order to understand if miRNA maturation of mir-1176 is a general pathway in *D. discoideum*, we also analyzed the biogenesis of the second model miRNA, mir-1177. As shown above, transcripts from the *mir-1177* gene accumulated in DrnB depleted cells in a manner similar to that of the *mir-1176* gene (Figure 3(b–e)).

#### 2.6.1. Transcriptional start site of pri-mir-1177

**Figure 5.**
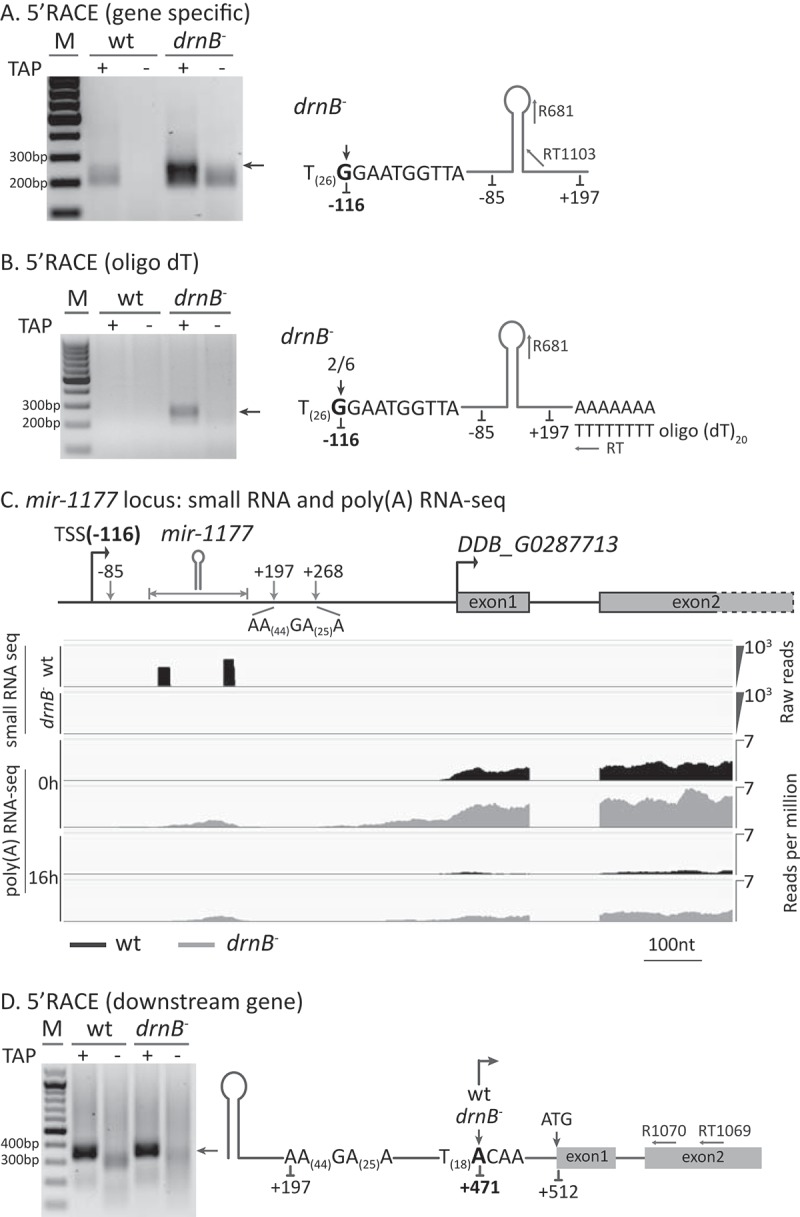
Pri-mir-1177 biogenesis in wt and *drnB*^-^ strains. (A,B,D) +/- TAP denotes RNA samples treated or untreated, respectively, with Tobacco Acid Pyrophosphates (TAP) prior to ligation of the RNA oligo. Arrows next to the gel pictures indicate PCR products that were isolated and sequenced. (A,B) 5ʹRACE to determine 5´-ends using gene specific and oligo(dT) primers, respectively, for reverse transcription. Schematic structures: oligonucleotides for reverse transcription and PCR (reverse primer) have prefixes RT and R, respectively. The 5ʹ most nucleotide determined by RACE and position in relation to the start of mir-5p are displayed as bold nucleotides and numbers. 5ʹ nucleotide from multiple RACE clones is indicated by numbers as fraction/total sequenced RACE-clones. Also, nucleotide positions for the very 5ʹ and 3´-ends of poly(A) RNA-seq reads are shown in regular font. (C) Small RNA-seq and poly(A) RNA-seq from wt and *drnB*^-^ strains. This is a modified version of Figure 3(c) emphasizing the region downstream of the miRNA hairpin. Top: schematic structure of the mir-1177 locus with the predicted miRNA hairpin indicated. TSS is the determined transcriptional start site. Numbers represent the TSS (bold) and mapped RNA reads covering the first part of the miRNA locus (−85 to + 197) and the poly(A) RNA reads starting after the A-rich genomic sequence (+ 268) in relation to the start of mir-5p. Exon1 and part of exon2 of the downstream protein coding gene are included. Panels just below the miRNA loci show the small RNA-seq results for wt (black) and *drnB*^-^ (grey). Small RNA-seq: for each strain, reads from two biological replicates from growing and developing cells, respectively, were pooled and are displayed as total read counts. Poly(A) RNA-seq: reads per million for pooled biological replicates from growing (0h) and developing (16h) cells, respectively, from wt (black) and *drnB*^-^ (grey). (D) 5ʹRACE to determine the TSS of the downstream gene (only exons 1 and 2 are indicated). The most likely TSS is indicated in bold. The position of the primers for reverse transcription (RT) and reverse PCR primer (R) are indicated.

The 5´-end of the *mir-1177* gene, mapped by poly(A) selected RNA reads, is positioned 85 nt upstream of the mir-1177-5p (Figure 3(c)). However, we reasoned that this cannot be the first nucleotide of pri-mir-1177 due to the way the RNA-seq libraries were generated (cap-structures prevent cloning of reads covering TSSs) and the fact that RT-PCR indicated that the pri-miRNA starts further upstream of this position (Figure 3(e)). To determine the TSS of pri-mir-1177, 5´RACE was performed similarly to the analysis of pri-mir-1176. First, we conducted 5´RACE where the cDNA was generated from a primer placed downstream of the predicted mir-1177 hairpin. The result showed two differently sized PCR-products where the larger one could only be detected in TAP-treated samples from *drnB*^-^ cells, indicating enrichment of capped pri-miRNA in the absence of DrnB (Figure 5(a)). The 5´RACE result is in agreement with the stabilization of longer transcripts from the *mir-1177* gene observed by poly(A) RNA-seq (Figure 3(c)) and semi-qRT-PCR (Figure 3(e)). Sequencing of these TAP-dependent 5´RACE products showed that, similar to pri-mir-1176, the 5ʹ most end of pri-mir-1177 starts with a G preceded by a run of T residues (26 consecutive Ts). The G residue, defining the very 5´ end, is situated 116 nt upstream of the start of mir-1177-5p (Figure 5(a)). The additional sequenced 5'ends indicated slightly shorter fragments, mainly within 19 nt from the 5ʹ most G that may constitute additional TSSs (Fig. S5A). The smaller 5ʹRACE products were detected in all samples except for those not treated with TAP from wt cells (Figure 5(a)). However, we cloned and sequenced a few of these RACE products generated from TAP treated wt cells and they turned out to derive from unrelated RNA due to mispriming (data not shown).

When 5´RACE was performed on oligo(dT)-selected RNA, only one prominent 5´RACE product was detected, from *drnB*^-^ TAP-treated cells (Figure 5(b)). Sequencing results showed a small enrichment of 5´-ends starting with the same G residue at position 116 (two out of six clones), while the remaining four clones coincided with the 5´ ends identified by the gene specific 5´RACE above (Fig. S5B). To summarize, our data suggest that pri-mir-1177 starts with a capped G residue 116 nt upstream of mir-1177-5p and that this TSS is preceded by 26 consecutive T residues.

#### 2.6.2. Stabilization of pri-mir-1177 in DrnB depleted cells reveals transcripts extending into the downstream protein coding gene

The start codon of a protein coding gene, DDB_G0287713 of unknown function (dictybase.org), is situated only 361 nt downstream of the predicted mir-1177 stem-loop (Figure 5(c)). For simplicity, from here on we will refer to this gene as the downstream gene (DSG). The pri-mir-1177 as well as the DSG mRNA appear to be upregulated in the *drnB*^-^ strain, both during growth and development (Figure 3(c,e) and 5(c)). Interestingly, reads from the *drnB*^-^ poly(A) RNA-seq libraries covered the entire region, starting upstream of the mir-1177 stem-loop and extending into the three exons of DSG, only interrupted by a long near consecutive stretch of A residues situated in between the miRNA hairpin and the start codon of DSG (Figures 3(c) and 5(c)) To simplify, only exon 1 and 2 of DSG are shown in the Figure 5(c). The lack of reads from the run of As is most likely due to sequencing difficulties and/or that the reads were discarded during computational analysis.

The extended reads that cover both genes raised the question if pri-mir-1177 is part of an unusually long 5ʹ untranslated region (5ʹUTR) or if both genes have individual promoters and hence, that transcription of pri-mir-1177 merely continues into the DSG, at least in the absence of DrnB. To address this, we performed RT-PCR experiments where primers were designed to reveal the identity of different transcripts covering the pri-mir-1177 and the DSG loci (Fig. S5C and Supplementary Extended Result). The results indeed show that pri-mir-1177 transcription reads into the DSG, supporting the poly(A) RNA-seq data in Figure 5(c). These transcripts appears to be stabilized in DrnB depleted cells, which is most likely due to the absence of Dicer dependent processing of the miRNA. Furthermore, the RT-PCR results also indicate that the increase in reads covering the DSG as compared to the *mir-1177* gene (Figure 5(c)) is due to transcription from a putative DSG promoter and that these transcripts are not affected by DrnB (Fig. S5C). Taken together, these results suggest that transcription of pri-mir-1177 continues into the DSG and that these transcripts accumulate in cells depleted of DrnB. Furthermore, transcripts controlled by the putative DSG promoter alone appears to be unaffected by DrnB.

#### 2.6.3. Independent promoters for pri-mir-1177 and the downstream gene

The data so far suggest that pri-mir-1177 is not part of the 5ʹUTR of the DSG and hence, the two genes are most likely transcribed from their own individual promoters. In order to confirm the presence of distinct promoters for the pri-mir-1177 readthrough transcripts and the DSG, we performed 5´RACE on +/-TAP treated RNA. In contrast to the previous experiment (Figure 5(a,b)), we now used the gene specific primer (RT1069) binding to exon 2 of the DSG for reverse transcription. Hence, this will generate cDNAs for both pri-mir-1177 readthrough transcripts as well as for DSG specific transcripts. We first analyzed the TSS of pri-mir-1177 readthrough transcripts. To this end, PCR amplification of the 5´-ends was performed as describe above (Figure 5(a,b)) and the same sized PCR products were observed from TAP-treated samples from *drnB*^-^ cells (Fig. S5D). This shows that TSS of pri-mir-1177 readthrough transcripts coincide with the above identified start of transcription and indicate stabilization of these capped transcripts in DrnB depleted cells.

Next, we performed a similar analysis (using the same cDNA) to identify the putative TSS of the DSG. This time the downstream PCR primer (R1070) was placed in exon 1 of the DSG. This generated distinct amplification products from TAP-treated RNA isolated from both wt and *drnB*^-^ cells (Figure 5(d)). The size of the fragment, ~350 bp, indicated that transcription of DSG starts around 50 bp upstream of the ATG start codon. In addition, although not strictly quantitative, the amount of PCR products was similar in samples from wt and *drnB*^-^ cells suggesting that DSG transcripts are not affected by DrnB. This is in line with the result from the RT-PCR experiment shown in figure S5C. Sequencing of the TAP dependent PCR products from both wt and *drnB*^-^ cells, revealed a few 5´-ends in close vicinity where the 5ʹ most nucleotide is an A-residue 42 bp upstream of the start codon (Figure 5(d) and S5E). The identified TSS for DDB_G0287713 is further corroborated by the poly-T tract immediately upstream, a feature of many protein coding genes in *D. discoideum* [51]. In conclusion, pri-mir-1177 is not merely part of a long 5ʹUTR belonging to the DSG. Instead, both genes are transcribed from individual promoters and transcripts from the DSG promoter appear unaffected by DrnB.

### 2.7. Primary transcripts of mir-1176 and mir-1177 carry the RNA polymerase II specific m^7^G-cap.

In animals and plants, most pri-miRNAs are transcribed by RNA polymerase II [4,5]. This may also be the case for pri-miRNAs in *D. discoideum* since mir-1176 and mir-1177 disappear in nuclear run-on experiments subjected to α-amanitin treatment [30]. Consistently, the predicted TSS of pri-mir-1176 and 1177 can only be detected by 5´RACE after TAP treatment (Figures 4(a,e) and 5(a,b)), indicating that these transcripts are capped. In order to verify that the 5´-ends of the pri-miRNAs have a bona fide RNA Pol II cap structure, we isolated 7-methyl guanosine (m^7^G)-capped RNAs using variants of the eukaryotic translation initiator factor 4E (elF4E). This protein has high affinity for 5´ m^7^G capped RNA Pol II transcripts and has been used to specifically pull down mRNAs, pri-miRNAs, and non-canonical capped pre-miRNAs from mammalian cells [4,52,53].

**Figure 6.**
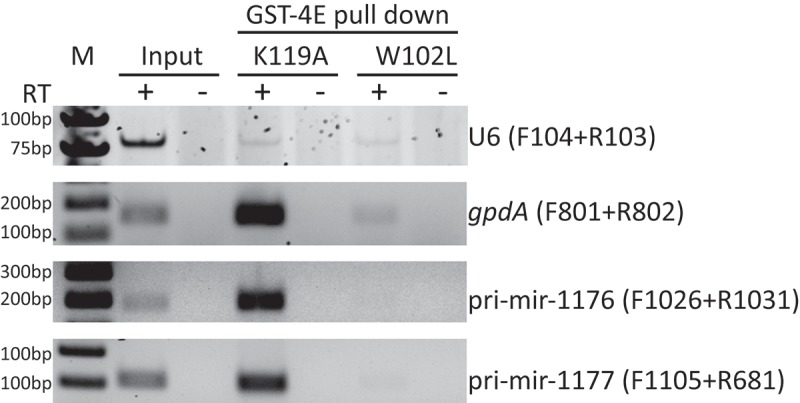
Pri-miRNAs have RNA Pol II specific cap-structures. GST tagged variants of eIF4E, GST-4E K119A and GST-4E W102L with increased or deficient binding affinity, respectively, for m^7^G capped RNA were used to analyze pri-miRNA cap structures, U6 spliceosomal RNA (negative control), and *gdpA* mRNA (positive controls). Equal quantity of RNA from input and GST-4E pulled down fractions were used for RT-PCR where reverse transcriptase (RT) was included (+) or excluded (-). Primers used are indicated to the right.

Glutathione S-transferase (GST) tagged elF4e mutant proteins, GST-4E W102L or GST-4E K119A, with deficient or increased cap binding affinity, respectively [52–54], were expressed in *Escherichia coli* (Fig. S6) and bound to glutathione magnetic beads followed by incubation with total RNA isolated from *drnB*^-^ cells. RT-PCR analysis demonstrated that both pri-mir-1176 and pri-mir-1177 were enriched by elF4E K119A but not by W102L (Figure 6). As expected, the positive control, *gdpA* mRNA, showed the same enrichment pattern as the pri-miRNAs. In contrast, the negative control U6 snRNA, which is transcribed by RNA Pol III and lacks the 5´ m^7^G cap-structure [55,56] did not show specific affinity for either elF4E K119A or W102A. We conclude that mir-1176 and mir-1177 primary transcripts have 5´m^7^G-cap structures, strongly supporting that they are transcribed by RNA Pol II.

### 2.8. Conserved motif upstream of pri-miRNA transcriptional start sites

**Figure 7.**
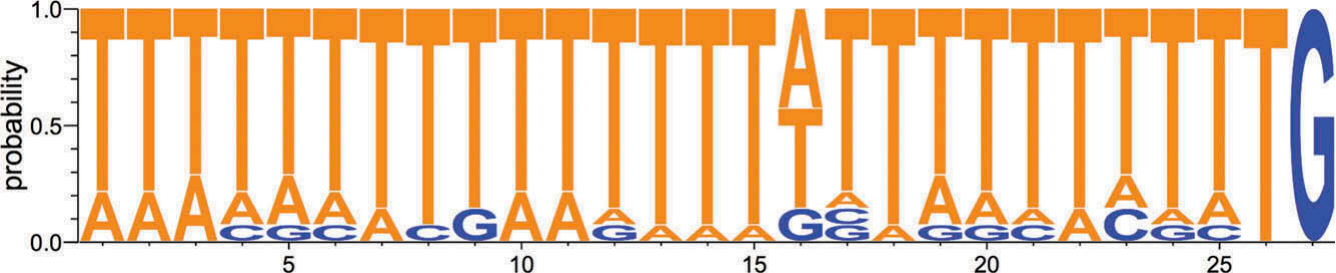
The pri-miRNA TSS motif. Nucleotide frequency per position was analyzed for conserved motif upstream of 14 *D. discoideum* pri-miRNA loci.

We hypothesized that the discovery of new miRNAs in this study, together with the previously identified miRNAs [22,28,29] would allow for a systematic search for common motifs defining the TSSs of pri-miRNAs. The analyses of pri-mir-1176 and 1177 show that these pri-miRNAs start with a G-residue situated 281 nt and 116 nt, respectively, upstream of the 5´-end of mir-5ps and that the TSSs are preceded by runs of T-residues. In addition, poly(A) RNA-seq reads, at least from *drnB*^-^ cells, cover the region between the predicted miRNA hairpins and the TSSs (Figure 3(b,c)). These experimentally defined features were used to search for putative TSSs of miRNA loci reported in this and previous studies [28,29]. For this analysis we excluded miRNA loci placed in introns, the multicopy miRNA loci that are part of the thug-S repetitive element [28], and the loci for which no reads were present in the RNA-seq libraries. Based on these criteria, we could predict a putative TSS for 14 pri-miRNAs (including pri-mir-1176 and 1177) consisting of a G-residue preceded by sequences of up to 26 nt heavily enriched for T-residues. This motif is present within ~140 nt from the 5ʹ most poly(A) RNA seq read (Figure 7). Similarly to pri-mir-1176 and 1177, we experimentally confirmed that also transcription of pri-mir-7097 starts downstream of the conserved T-rich motif (Fig. S7). The miRNA loci used for this analysis and their respective motif can be found in supplemental table S3.

### 2.9. Discussion

In contrast to animal and plants where miRNAs are prevalent, miRNAs have only been identified in a few unicellular eukaryotes [27] and details about their biogenesis are very sparse. For example, in the supergroup Amoebozoa, high confidence miRNAs have only been identified in *D. discoideum* [28]. Hence, an important question is how miRNAs and their associated factors have evolved. Do miRNA and their production share characteristics with plants and/or animals or have they evolved specific features adapted for unicellular lifestyles? In order to gain further insight into the miRNA machinery in an unicellular organism, we investigated the global effect of the Dicer-like protein DrnB on small RNAs maturation in the social amoeba *D. discoideum*, with an emphasis on miRNAs and their biogenesis. Since *D. discoideum* is unicellular during growth but develops as a multicellular organism and our previous studies have shown that miRNAs in *D. discoideum* are developmentally regulated [22,28], we included both growing single cells and the multicellular slug/finger stage in our study. Thus, DrnB dependent regulation could be investigated during both life stages.

Strikingly, sequencing of small RNAs from wt and DrnB depleted cells revealed a dramatic reduction of miRNAs in the latter strain, demonstrating the importance of DrnB in miRNA biogenesis in *D. discoideum*. This is in line with previous results where northern blot analyses showed that a few miRNAs are DrnB dependent [22,28,29]. No major effect of DrnB was observed for other small RNAs, including siRNAs derived from DIRS-1, which constitute ~ 68% of the small RNA population in both wt and *drnB* knock-out cells. This strongly suggests that DrnB is dedicated to miRNA production and that the other Dicer-like protein DrnA, present in *D. discoideum* [48], is required for generating siRNAs. Similar division of functionality between Dicer/Dicer-like proteins is also seen in other organisms such as plants and the two Dicers present in Drosophila where Dicer-1 and Dicer-2 are devoted to miRNA and siRNA maturation, respectively [6,57,58]. However, in the single cell alga *C. reinhardtii*, one of the three Dicer homologs, DCL-3 is required for both miRNA and siRNA maturation, a situation similar to mammals and worms where a single Dicer protein produce both classes of small RNAs [31,57]. It should be noted that a small proportion of the small RNAs, derived from the LTR retrotransposon TRE-3A [41], for unknown reasons accumulate in *drnB*^-^ cells.

The small RNA-seq from unicellular growing cells and multicellular developing structures from wt and *drnB*^-^ *D. discoideum* allowed us to identify ten new DrnB dependent miRNAs in *D. discoideum*. Two (miRNA_can_D1 and miRNA_can_D2) of the four miRNAs recently reported by Meier *et al*., also fulfilled our stringent search criteria, which adds up to 29 miRNAs discovered in *D. discoideum* [28,29]. In line with our previous results [28], all these new miRNAs are developmentally regulated. The majority of the miRNAs in *D. discoideum* appears to derive from intergenic loci except two, which are generated from intronic sequences of protein coding genes, corroborating the genomic sources of miRNAs previously reported by us [28]. Interestingly, the two intron sequences generate each two sets of miRNA pairs (mir-5p and mir-3p pairs) where each pair is situated immediately adjacent to each other. This resembles some of the miRNA-like RNAs in plants and the miRNA-offset RNAs (moRs) present in animals, where more than one miRNA derive from the same pre-miRNAs [32,33,47]. Although miRNAs are severely reduced in the *drnB* knock-out strain, some small RNAs, including miRNAs, derived from the miRNA hairpin precursors are still present (Fig. S2). A similar situation is observed when Dicer and DCL1 are depleted in human cell-lines and *Arabidopsis thaliana*, respectively [59–61]. We speculate that the presence of low levels of miRNAs in DrnB depleted cells is due to processing by DrnA. This is supported by the increased small RNA heterogeneity observed in the absence of DrnB indicative of DrnA RNase III activity (Fig. S2). These findings, together with our result showing that small RNA population matching both strands of the DIRS-1 is not affected in DrnB depleted cells (that still express DrnA) strongly indicate that DrnA and DrnB are functionally separated, dedicated to siRNA and miRNA production, respectively. It should be noted that in spite of several attempts in different laboratories, ours included, the gene for *drnA* could not be knocked out indicating an essential role for this RNase III enzyme.

The observed dependency of DrnB for miRNA maturation suggested that pri-miRNA and possible processing intermediates could be accumulating in the *drnB* knock-out strain. This was confirmed by our poly(A) RNA-seq data showing a dramatic increase of RNA covering the miRNA loci in DrnB depleted cells as compared to wt cells. This is in line with a recent study where longer transcripts from the *mir-1176* and *1177* genes accumulate when the genes encoding DrnB and RbdB, forming the microprocessor complex in *D. discoideum*, are disrupted [29]. Thus, we reasoned that by taking advantage of the DrnB-dependency on miRNA processing, i.e. comparing pri/pre-miRNA transcripts from wt and *drnB*^-^ cells, we should be able to get insight into miRNA biogenesis pathway(s) in *D. discoideum*. To this end, we analyzed two of the most abundant miRNAs in *D. discoideum*, mir-1176 and mir-1177.

The TSS for both pri-mir-1176 and 1177 were mapped to capped G-residues 281 nt and 116 nt, respectively, from the start of their mir-5ps embedded in downstream hairpin structures. Moreover, for each genomic location these G residues are preceded by a stretch of Ts, i.e. 21 nt and 26 nt Ts for pri-miRNA 1176 and 1177, respectively. This appears to be a general sequence feature associated with miRNA loci in *D. discoideum* since similar motifs are present in close vicinity to the 5ʹ most poly(A) RNA-seq reads of the 14 intergenic miRNA genes. Poly-T tracts immediately upstream of TSSs is a common feature for protein coding genes in *D. discoideum* [51], indicating that also *D. discoideum* pri-miRNAs are transcribed by RNA pol II. This idea is also supported by a previous study by Kruse *et al*., who showed reduction of mir-1176 and mir-1177 upon treatment with α-amanitin [30] and is in agreement with the situation in animals and plants where most pri-miRNAs are transcribed by RNA Pol II [4–6]. Furthermore, mammalian RNA Pol II transcripts preferentially start with a purine preceded by a pyrimidine [53,62], which complies with the T and G at position −1 and +1, respectively, seen for the pri-miRNA associated motif in *D. discoideum*. This purine-pyrimidine preference was also reported for miRNA genes in Arabidopsis [5]. We further corroborated that pri-miRNAs, i.e. pri-mir-1176 and pri-mir-1177, are bona fide RNA Pol II transcripts by showing that they are specifically pulled down by active elF4e that recognize the RNA Pol II specific m^7^G-cap structures. Hence, RNA Pol II transcription of miRNA genes appears to be a conserved feature, present in the unicellular amoeba *D. discoideum* as well as in multicellular animals and plants [4,5].

**Figure 8.**
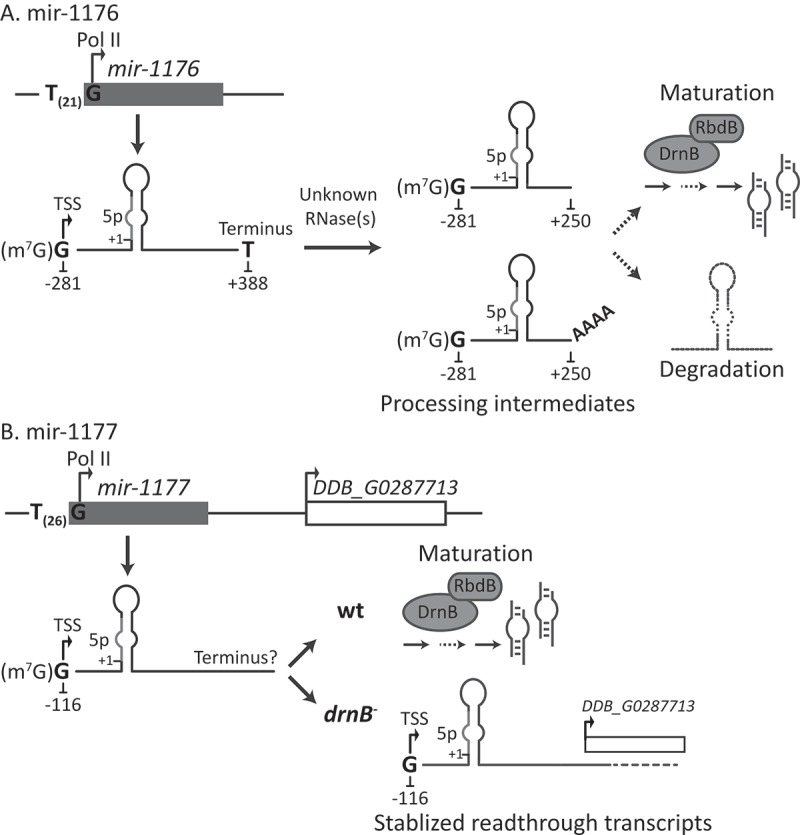
Identified microRNA biogenesis pathways in *D. discoideum*. The picture show models for biogenesis for mir-1176 and mir-1177. (A,B) Both pri-mir-1176 and 1177 are transcribed by RNA Pol II where the TSSs begin with G residues preceded by runs of T nucleotides, 21 nt and 26 nt, respectively. (A) Pri-mir-1176 terminates 388 nt from the start of mir-1176-5p. These non-polyadenylated transcripts are further processed by unknown RNase(s) to shorter precursors of which some carries a post-transcriptionally added A-tail. The precursors may be a direct target for the microprocessor (DrnB and RbdB) where DrnB is responsible for generating the mature miRNAs. The role of the A-tail is not known but perhaps it tags precursors, not processed by the microprocessor, for degradation. (B) The terminus for pri-mir-1177 has not been determined and shorter precursors were not detected, indicating a rapid maturation of miRNAs by the microprocessor in wt cells. In DrnB depleted cells, stabilized pri-miRNA transcripts extend into the downstream gene.

In addition to the 5ʹ-ends of pri-mir-1176 and pri-mir-1177, we analyzed formation of their 3ʹ-ends. In the case of pri-mir-1177, the stabilized pri-miRNA seen in DrnB depleted cells is extended into the downstream gene. We demonstrate that the pri-mir-1177 does not simply reside in the 5´ UTR of the downstream gene since both genes have individual TSSs. Furthermore, the two genes show different expression levels and the pri-miRNA is dramatically stabilized in *drnB*^-^ cells while transcripts of the downstream gene is less, if at all, affected by DrnB. One possibility that could explain the accumulation of readthrough transcripts of pri-mir-1177 in *drnB*^-^ cells is that DrnB normally assists in transcriptional termination of the pri-miRNA followed by rapid miRNA maturation also performed by DrnB. This is reminiscent of a recently proposed mechanism where the microprocessor in human cells induce cleavage and thereby termination of lncRNAs and hence, when components of the microprocessor were depleted, extensive readthrough of pri-miRNAs was observed [63], similar to what we see for pri-mir-1177 transcripts in *drnB*^-^ cells. Interestingly, 3´-end formation of pri-mir-1176 appears very different from pri-mir-1177. In wt cells, a distinct termination is detected 388 nt downstream of the mir-1176-5p. Even though our data strongly suggest that miRNA genes in *D. discoideum* are transcribed by RNA Pol II, the 3´-end of pri-mir-1176 detected in wt cells is not polyadenylated and no canonical polyadenylation signal is present. This is in contrast to the polyA-tailed canonical pri-miRNAs in plants and animals [5,49,64]. However, a study on human cells reported that long noncoding (lnc)RNAs hosting miRNAs are not subjected to the canonical polyadenylation pathway [63]. Our results further indicate that the 3´-end of pri-mir-1176 is processed by unknown ribonuclease(s) into a few shorter intermediates, stabilized in cells depleted for DrnB. A fraction of these carries a short untemplated A-tail in both *drnB*^-^ and wt cells (A-tails in the latter was only observed by specific selection of A-tailed RNAs using oligo(dT) RT-PCR). We speculate that the processing intermediates, stabilized in *drnB*^-^ cells, normally are further processed by DrnB to mature miRNAs. If for some reason the precursor-miRNAs are not cleaved by DrnB, short A-tails may be added to their 3´-ends and target them for degradation. This may be orchestrated by the conserved TRAMP polyadenylation complex together with the exosome. The TRAMP complex is involved in adding short A-tails to e.g. misfolded or improperly processed ncRNAs such as snRNA and tRNAs and thereby target them for degradation by the exosome [65,66]. In Arabidopsis, TRAMP-like and exosome activities have been suggested to be responsible for oligoadenylation and subsequent degradation of intermediates of miRNA biogenesis. However, in contrast to mir-1176 precursors, these short A-tracts were added upstream of the stem-loop harboring the mir-5p/3p duplex [67]. We have previously shown that short A-tails are present in a fraction of the snRNAs as well as on rRNA precursors in *D. discoideum* [55,68], suggesting that oligoadenylation is a common feature also in *D. discoideum*. Furthermore, gene ortholog for the components of both TRAMP and the exosome are present in *D. discoideum* [69]. Even though we find it plausible that A-tailing of processing intermediates of pri-mir-1176 target them for destruction and thereby lead to recycling of the components of the miRNA machinery, at this point we cannot rule out that they may be processing intermediates targeted by DrnB to generate mature miRNAs. Models for miRNA biogenesis pathways of mir-1176 and 1177 are illustrated in Figure 8.

The origin of miRNA pathways have been discussed many times and the prevailing belief is that miRNAs evolved independently several times during the course of evolution although this idea of convergent evolution has recently been challenged [6,27,70,71]. The phylogenetic position of *D. discoideum* within the Amoebozoa supergroup, which branched off after plants (Archaeplastida) but before animals (Opisthokonta) [72], gives us glimpses into the evolution of the miRNA machinery. The miRNA pathway in *D. discoideum* share several features with plants. For example, we show that the majority of the miRNAs derive from lncRNAs, although shorter than in plants presumably due to the compact genome of *D. discoideum* [26], and that miRNAs generated from introns of protein coding genes are rare. Furthermore, similar to the miRNA processing Dicer-like protein in plants, DrnB is present in the nucleus [73] where it seems to be responsible for both generating pre-miRNA and for maturation of the mir-5p and −3p. In contrast, animals carry Drosha, which acts in the nucleus where it cleaves pri-miRNA to pre-miRNA, which is further processed by cytoplasmic Dicer to miRNA-duplexes [10,74]. No obvious homolog to Drosha is present in *D. discoideum* eventhough the domain structure of DrnB is similar[30]. Interestingly, this Drosha-like domain arrangement is also present in in DCL-3, the Dicer protein responsible for miRNA maturation in the unicellular alga *C. reinhardtii* [31]. A feature of the miRNA machinery in *D. discoideum* shared with animals is the absence of 3ʹ methylation of miRNAs. We have previously shown that at least one of the miRNAs in *D. discoideum*, mir-1177, lack 2´-O-methyl modification at the 3ʹ-end [28]. The same is true for metazoan miRNAs while plant miRNAs carry this protective methyl group at their 3ʹ-ends [57]. Finally, the short A-tails that we see on processing intermediates of mir-1176 transcripts may be a *D. discoideum* specific adaptation to regulate pre-miRNA turnover and thereby the levels of miRNAs in the cell. This would be somewhat analogous to the situation in animals, where oligo-uridylation of the let-7 miRNA precursor inhibit Dicer processing and induce degradation [75].

Whether the composite nature of the miRNA machinery in *D. discoideum*, with characteristics from both plants and animals but also unique features, is due to adaptation to its specific lifestyle, unicellular growth and multicellular development, or an evolutionary consequence of its intermediate phylogenetic position is presently not known. Further studies of miRNAs in this intriguing organism will be required to answer this question.

## 3. Materials and methods

### 3.1. Cells, growth conditions, and development

*D. discoideum* AX2 wild-type (wt) and *drnB* knock-out (*drnB*^-^) cells were grown axenically in HL5 medium supplemented with 10 μl/mL Penicillin Streptomycin (ThermoFisher Scientific) at 22°C. For each development, approximately 5 × 10^7^ cells were collected and washed twice with PDF buffer (20mM KCl, 5mM MgCl_2_, 20mM KPO_4_, pH at 6.2), resuspended in 500 µl PDF buffer and spread on a nitrocellulose membrane (Millipore) followed by incubation at 22°C [76].

### 3.2. Oligonucleotides

DNA and RNA oligonucleotides, listed in table S2, were synthesized by Sigma-Aldrich and Dharmacon, respectively.

### 3.3. RNA isolation

Total RNA was isolated from 1 × 10^8^ cells from growing cells and from developing stages, i.e. 16h slugs and 24h fruiting bodies, using TRIzol (ThermoFisher Scientific) according to the manufacturer’s recommendation.

### 3.4. High-throughput sequencing

Small RNA and poly(A)-selected RNA-sequencing was carried out by SciLifeLab Stockholm. Briefly, biological duplicates of total RNA isolated from wt and *drnB*^-^ cells during growth (0h) and development (16h) was converted to cDNA and sequenced using the Illumina platform. For poly(A)-selected RNA-seq, RNA samples were purified by poly(A)-enrichment before libraries were created using TruSeq RNA Library Prep Kit (Illumina). TruSeq Small RNA Library Preparation Kit (Illumina) was used to construct strand specific small RNA libraries (18–40 nt). Poly(A)-selected RNA libraries were sequenced using Illumina HiSeq-2500 (50 bp single reads) and small RNA libraries were sequenced using Illumina HiSeq-2000 (100 bp single reads). The sequence data have been submitted to the DDBJ/EMBL/GenBank databases under accession number GSE111592.

### 3.5. Bioinformatic analyses

Genomic sequence [26] and annotations (generated June 3, 2015) were retrieved from www.dictybase.org. Mapping of poly(A)-selected reads was performed with TopHat v. 2.0.8b [77] using default settings. Read counts were generated with HTSeq-count v. 0.6.1p1 [78] and differential expression analysis was performed using DESeq2 package v. 1.8.1 [79]. Coverage of RNA reads were analyzed and visualized with Integrative Genomics viewer [80,81]. Small RNA sequencing reads (≥18nt) were mapped to the genome sequence using Bowtie 1.1.1 [82] allowing for two mismatches. miRNA prediction were performed with miRDeep2 [43], ShortStack 3.6 [44] and the pipeline for high-throughput identification of miRNAs in *D. discoideum* [28] using all wt small RNA sequencing data as input. Small RNA reads were mapped to the predicted miRNA hairpin-sequences and visualized using in-house python script. Structures and stability of candidate hairpins were predicted using mfold (version 2.3 energies) with folding temperature set to 22°C.

## 4. RT-PCR

RT-PCR in Figure 4(b) was performed using the same cDNA templates as for Figure 4(a) (5ʹRACE cDNA reverse-transcribed using RT1083, see below) and genomic DNA (g.DNA) as a positive control. The reactions were carried out with the same reverse primers (R1031) but different forward primers, which were placed upstream (F1025) and downstream (F1026) of the identified transcriptional start site. Similar analyses were performed to verify the predicted T-rich motif upstream of pri-mir-7097 in Figure S7A. In PCR reactions, forward primer F1218 and F1217, located upstream and immediately downstream of the predicted motif, respectively, were used together with the reverse primer R1221. PCR products were amplified from either 5ʹRACE cDNA templates synthesized using oligo dT (see below) or from g.DNA template. Cycling conditions for both PCRs were 95°C for 5 min; 35 cycles of [95°C for 30 s, 53°C for 30 s, 62°C for 45s] and 62°C for 5 min. The RT-PCR experiment presented in Figure S5C was performed as follows: 5 μg DNase I (ThermoFisher Scientific) treated RNA (from the same DNase I treated RNA used in RACE experiments below) each from wt and *drnB*^-^ strains was reverse transcribed into cDNA in 20 μl reactions at 55°C with 15 U ThermoScript™ Reverse Transcriptase (ThermoFisher scientific) and 20 μM primer RT1069. PCR analysis was carried out in 20 μl using DreamTaq Green DNA Polymerase (ThermoFisher Scientific) to which 1 μl of the cDNA template had been added. Primer pairs included in the separate reactions were F1066 and R1068, F1071 and R1070, and F1066 and R1070 to analyze pri-mir-1177, DSG, and the readthrough transcripts. Cycling conditions for pri-mir-1176 and DSG were as follows: 95°C for 5 min and 35 cycles of [95°C for 30s, 60°C for 30s, 62°C for 45s] followed by 5 min at 62°C. The extension time for analysis of the readthrough transcript was extended to 1.5 min due to the longer transcript.

### 4.1. Semi-quantitative RT-PCR

DNase I treated RNA (2,5μg each) from wt and *drnB*^-^ cells (0h and 16h) was reverse-transcribed into cDNA according to Maxima H Minus First Strand cDNA Synthesis Kit (ThermoFisher Scientific). Primers RT103 (20 μM), RT1083 (10μM), and RT1103 (10μM) were used for reverse transcription of snRNA U6 [55], pri-mir-1176, and pri-mir-1177. PCR reactions were carried out in 20μl PCR master mix (ThermoFisher Scientific) to which gene specific primers and 1μl of the corresponding cDNA was added. In brief, for amplification of the U6 reference control, primers F104 and RT103 was used. Cycling condition were 95°C for 5min and 10 cycles of [95°C for 30s, 60°C for 30s, 62°C for 15s]. To determine the optimal number of cycles (10 cycles) where amplification is exponential, the same PCR reaction was performed also with 5 and 15 cycles (Fig. S3). The U6 PCR products were visualized by 6% native polyacrylamide gel stained with SYBR Safe DNA gel stain (ThermoFisher Scientific). Primer pairs F1026+R1031 and F1105+R681 were used to quantify the expression of pri-mir-1176 and pri-mir-1177. The same PCR conditions were used for both pri-miRNAs, i.e. 95°C for 5 min; 35 cycles of [95°C for 30s, 53°C for 30s, 62°C for 45s] and a final extension at 62°C for 5 min. The PCR products were analyzed by agarose gel electrophoresis.

### 4.2. Stem-loop RT-QPCR

The analysis was performed essentially as described before [42]. Briefly, the method relies on a RT-primer forming a stem-loop structure but where the 5´-end is complementary to the 3´-end of the miRNA to be analyzed. After reverse transcription, the resulting cDNA sequence contains the reverse strand of the miRNA and at the 5´-end, a set sequence determined by the primer. This is then used as template for real-time PCR. cDNA synthesis was carried out using RevertAid First Strand cDNA Synthesis Kit (ThermoFisher Scientific) according to the manual but with minor modifications. Biological duplicates of DNase treated total RNA (1μg) were converted to cDNA in reactions containing 5 μM RT103 (U6), 1μM RT961 (mir-1177), 1μM RT963 (mir-1176). The reverse transcription reaction was incubated at 16°C for 30 min followed by 25°C for 5 min followed by the procedure described by the manufacturer. U6 was used as reference control. Real-time PCR was carried out using Step One Plus Real Time PCR System (Applied Biosystems) where 1 or 2μl of 5 times diluted cDNA was added to the qPCR reactions to analyze expression of U6 or mature miRNAs, respectively. Real-time quantification PCR was performed in 15μl reactions including 7.5μl Maxima SYBR Green/ROX qPCR Master Mix (ThermoFisher Scientific), and 5μM of primer pairs for amplification of U6 (F104 and R20), for mir-1176 (F964 and R969), and for mir-1177 (F962 and R969). The cycling condition was 95°C for 10 min followed by 40 cycles of [95°C for 15s, 60°C for 30s] followed by melting curve analysis using machine default settings. All reactions were performed in technical duplicates. The specificity of the primers (to generate correct PCR products) was confirmed by 6% native polyacrylamide gel electrophoresis as well as Sanger-sequencing (Macrogen).

### 4.3. 5ʹ RACE

5ʹRACE was carried out essentially as described previously [53] but with a few modifications. Briefly, RNA was extracted from 0h, 16h, and 24h cells and mixed at a ratio of 4:3:3, respectively. DNase treated RNA was dephosphorylated by calf intestinal alkaline phosphates (CIAP) (ThermoFisher Scientific) at 37°C for 1h followed by phenol/chloroform extraction and ethanol precipitation. Subsequently, 15 µg of dephosphorylated RNA was treated with 25 U Tobacco Acid Pyrophosphatase (TAP) (Epicentre/Illumina) at 37°C for one hour. After phenol phenol/chloroform extraction and ethanol precipitation, 5 µg of TAP-treated RNA was ligated to GeneRacer RNA oligo (20 µM) in 50 µl reactions with 40 U of T4 RNA ligase (ThermoFisher Scientific) at 16°C overnight. Reverse transcription was performed in 20 µl reactions with 15 U ThermoScript Reverse Transcriptase (ThermoFisher Scientific) and primers RT1083, RT1103, and RT1069 specific for pri-mir-1176, pri-mir-1177, and DSG at 50°C (55°C for primer RT1069) whereafter the cDNAs were treated with 5 U of RNase H (ThermoFisher Scientific). Similar procedure was employed for 5ʹRACE where 50 µM of oligo(dT)_20_ (ThermoFisher Scientific) was used for reverse transcription. However, the CIAP treatment was omitted. For both 5ʹRACE assays, control samples were treated under identical conditions but with the exclusion of TAP. The cDNAs were PCR amplified using GeneRacer 5ʹ primer and gene specific primers R1031, R681, R1070, and R1221 for pri-mir-1176, pri-mir-1177, DSG and pri-mir-7097. Cycling conditions for the pri–mir-1176 and −1177 were 95°C for 5 min; 35 cycles of [95°C for 30 s, 53°C for 30 s, 62°C for 1 min] and 62°C for 5 min. For amplifying 5ʹ-end of pri-mir-7097, similar cycling conditions were applied but with the annealing temperature of 57°C. The cycling conditions for DSG differed in that the annealing temperature was 60°C and the extension time was 1 min and 45 sec. In order to increase the amount of products, the PCRs were repeated using 1 µl of the reactions from the previous PCRs. The PCR products were analyzed by agarose gel electrophoresis. The sequences of the 5´-ends were determined by sequencing cloned PCR products.

### 4.4. 3ʹRACE

The DNA adapter (oligo ced37) (40 µM) blocked at its 3´-end by Spacer C3 modification, was ligated to the 3´-end of 6 µg DNase treated and dephosphorylated RNA as described for 5´RACE. The resulting product was split into two cDNA reactions where 100 µM oligo ced41, hybridizing to the adapter oligonucleotide, was added to both reactions but reverse transcriptase only to one reaction, followed by incubation for 1 h at 50°C. The reaction conditions were the same for 3ʹRACE where polyadenylated pri-miRNA was analyzed except that ligation of the DNA adapter to the 3´-end of RNA was bypassed and instead the RNA was converted to cDNA using GeneRacer oligo dT_(18)_. 3ʹRACE PCR products of adapter ligated RNA were obtained by using primers ced41 and F1023 and 1 µl cDNA template. Cycling conditions were 95°C for 5 min; 40 cycles of [95°C for 30s, 53°C for 30s, 62°C for 45s] and 62°C for 5 min. The PCR was repeated using 1 µl PCR product as template and primer F1022 instead of F1023. For amplification of 3ʹRACE oligo(dT) products, 1 µl cDNA template was amplified with primers GeneRacer 3ʹprimer and F1023 in a touch down PCR where the annealing temperature was lowered every 5 cycles (repeated 3 times), i.e. 95°C for 5 min; [95°C for 30s, 65/62/55°C for 30s, 62°C for 1.5 min] followed by 30 cycles of [95°C for 30s, 55°C for 30s, 62°C for 1.5 min] and 62°C for 5 min. The PCR was repeated using 1 µl PCR product with primer F1050 instead of F1023 with cycling conditions of 95°C for 5min; 40 cycles of [95°C for 30s, 57°C for 30s, 62°C for 1min] and 62°C for 5 min. The PCR products were analyzed by agarose gel electrophoresis. The sequences of the 5´-ends were determined by sequencing cloned PCR products.

### 4.5. m^7^G-capped RNA purification and RT-semi-qPCR analysis

Plasmids expressing GST tagged eIF4E mutants, either K119A or W102L (generous gifts from Profs Curt H. Hagedorn and Joan Steitz, respectively), were transformed into *E. coli* BL21 (DE3) [52–54]. Two single colonies from each transformation were used as biological duplicates for protein expression. Briefly, cells were grown at 37°C in M9ZB media with 100ug/mL ampicillin. When cells had grown to OD600 of 0.5–0.6, protein expression was induced by 0.4 mM IPTG for 3 h followed by sonication [54]. Supernatants were mixed with pre-washed Glutathione Magnetic Beads (ThermoFisher Scientific) and incubated at 4°C overnight. The binding was assessed by boiling to release the GST-elF4E variants whereafter aliquots of the supernatants were analyzed by Mini-PROTEAN TGX Stain-Free precast protein Gels (Bio-Rad) and stained with QC Colloidal Comassie Stain (Bio-Rad).

The GST-elF4E variants, bound to glutathione beads, were used to analyze the presence of a 7-methyl guanosine (m^7^) cap on RNA. Experimental conditions for binding capped-RNA to the GST-elF4E and subsequent washing steps were as described before [52]. Briefly, RNA was extracted from *drnB*^-^ cells from different life stages, i.e. 0h, 16h and 24h and combined. The RNA (150 μg for each protein) was mixed with GST-4E W102L and GST-4E K119A bound to glutathione beads (300 μl volume) and incubated for 2 h at 4°C. Different from the protocol used by [52], RNA was eluted by one volume of phenol and incubation on ice for 15 min, followed by phenol/chloroform extraction and precipitation with ethanol. After DNase treatment, 584ng RNA purified from GST-4E pull-down and RNA input control (treated as described above but not mixed with GST-4E beads) was subjected to reverse transcription essentially as described for semi-quantitative RT-PCR above but with different primers, i.e. RT103 for U6, RT975 for *gpdA*, RT1083 for pri-mir-1176, and RT1103 for pri-mir-1177. This was followed by PCR amplification as described for semi-quantitative RT-PCR. For amplification of the positive control *gpdA*, PCR was performed with primers F801 and R802 where cycling conditions were 95°C for 5 min and 25 cycles of [95°C for 30s, 60°C for 30s, 62°C for 30s]. U6 PCR products were analyzed on 6% native polyacrylamide gel whereas the pri-miRNAs and the *gpdA* products were analyzed by agarose gel electrophoresis.

### 4.6. Northern blot

Northern blots to verify newly discovered miRNA candidates were performed essentially as previously described [83]. Briefly, 20 μg total RNA was separated on 12.5% denaturing polyacrylamide gels and transferred to Hybond N+ nylon membranes (GE Healthcare). RNA molecules were immobilized on membranes by UV crosslinking and hybridized with oligonucleotide probes against U6 and miRNAs individually. U6 loading control: oligo 102 was used as probe and labeled by DIG at the 3´-end using DIG Oligonucleotide 3ʹ-End Labeling Kit, 2nd generation (Merck). Hybridizations were performed in standard buffer (5x SSC, 0.1% (w/v) N-lauroylsarcosine, 0.02% (w/v) SDS, 1% Blocking Reagent (Merck)) at 60°C overnight. Subsequently, membranes were washed and hybridization signals were analyzed by ChemiDoc Imaging System (Bio-Rad) according to the DIG System and the DIG Application Manual (Merck). The membranes were reprobed with ^32^P-end-labeled oligonucleotides 1121 and 885 for mir-1185-3p and mir-1183-5p as previously described whereafter hybridization was analyzed by PhospImager (Bio-Rad). GeneRule Ultra Low Range DNA Ladder (ThermoFisher Scientific) was labeled the same way as labeling oligo 102 and used as size standard.
