# Supplementary material for: Global characterization of the Dicer-like protein DrnB roles in miRNA biogenesis in the social amoeba Dictyostelium discoideum
Source: RNA Biol. 2018 Aug 21;15(7):937–54. doi: 10.1080/15476286.2018.1481697 (PMC6161686; doi:10.1080/15476286.2018.1481697)
Supplement: Supplemental Material [file krnb-15-07-1481697-s001.pdf]

Supplementary Extended Results

Stabilization of pri-mir-1177 in DrnB depleted cells reveals transcripts extending into the downstream gene.

The poly(A)-selected RNA-seq data showed extended reads covering both the *mir-1177* gene and the downstream gene (DSG) (Fig. 5C). This could be due to that pri-mir-1177 is part of the 5’UTR of the DSG or that the two genes have separate promoters and pri-mir-1177 transcription reads into the DSG. In order to answer these questions we first confirmed the poly(A) RNA-seq data indicating long transcripts covering both the mir-1177 and the DSG loci (Fig. 5C). For this, we used RT-PCR where the primer for cDNA synthesis, RT1069, hybridized to exon 2 of the DSG mRNA (Fig. S5C). The resulting cDNA was used as template for different PCR amplifications to reveal the origin of the transcripts. First, we analyzed if the predicted mir-1177 stem-loop is part of the extended transcripts (primers F1066 and R1068). This stem-loop is expressed as part of the pri-miRNA and hence should be amplified if it is also part of a longer transcript reading into the DSG. The PCR result showed products of expected size both when RNA from wt and *drnB*$^{-}$ cells was used for cDNA synthesis but with a marked increase in *drnB*$^{-}$ cells (Fig. S5C). This indicates that the predicted stem-loop, from which mir-1177 is derived, is part of a longer transcription unit that reads into the DSG and is destabilized in the presence of DrnB. Similar results were obtained when PCR primers, F1066 and R1070, were placed in the miRNA-stem-loop and in exon 2 of the DSG, respectively (Fig. S5C). These results, although not strictly quantifiable, are in line with the RNA-seq data indicating

readthrough of pri-mir-1177 into the DSG and that these transcripts accumulate in cells depleted of DrnB.

When the *mir-1177* gene is compared to the DSG, poly(A) RNA-seq data show a pronounced increase in reads matching the latter. This increase begins shortly upstream of the start codon for DSG (Fig. 5C). In order to analyze these transcripts, we performed RT-PCR using the same cDNA as above as template but this time PCR primers (F1071 and R1070) were designed to amplify cDNA covering the beginning of the open reading frame (ORF), i.e. parts of exon 1 and 2 of DSG. In contrast to the previous RT-PCR, this analysis would give us insight not only into the transcripts contributed by the pri-mir-1177 alone but also transcripts generated from a putative DSG promoter. Interestingly, contrary to the RT-PCRs indicating a distinct accumulation of pri-mir-1177 readthrough transcripts in DrnB depleted cells, transcripts covering exons 1 and 2 appear less affected by the absence of DrnB (Fig. S5C). This suggests that the main contribution of transcripts covering DSG, is not due to readthrough of pri-mir-1177 but rather DSG-specific transcripts that are not significantly affected by DrnB. Another observation from the RNA-seq data was the lack of reads matching the intron sequences between exon 1 and 2 (Fig 5C), showing that pri-mir-1177, like the DSG transcript, is spliced like a canonical RNA Pol II transcript. Taken together, transcription of pri-mir-1177 continues into the downstream gene and these transcripts are spliced and accumulate in cells depleted of DrnB. Transcripts controlled by the putative DSG promoter alone appears to be unaffected by DrnB.

Supplementary Figures and Legends

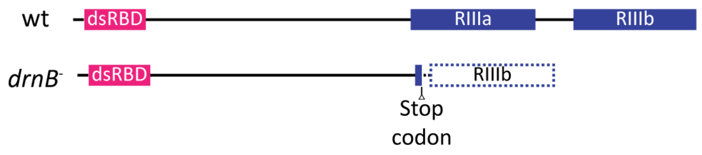

Supplementary Figure S1. Schematic picture of Dicer-like protein DrnB and the *drnB* knock-out construct (*drnB*$^{-}$). dsRBD: dsRNA-binding domain; RIIIa and RIIIb: RNase III domains. Dashed rectangle depicts the RIIIb domain remaining in the knock out construct, preceded by a stop codon introduced by the disruption vector (Avesson et al., 2012).

miRNA: mir-1178

WT	Pooled			0h		16h	
	total	mir-5p	mir-3p	mir-5p	mir-3p	mir-5p	mir-3p
raw	157	140	9	51	1	89	8
rpm	1.8	1.6	0.1	1.41	0.03	1.84	0.19

ATATTCTTAACAATACATTTTATTTTAAAAAAAATGGTATTAATTTGTAATATAAAAGATGAATTTGATTTGTTTATTAGATTAATAATTAAATCAAATTCATCTTTTATATTATAAATTAGTACTATTTTTTTTTTTAAAAAAAAAAGTACAAGTTACAATAT	count	length
--**TAATTTGTAATATAAAAGATG**---	139	21
--TAATTTGTAATATAAAAGA---	1	19
--TAATTTGTAATATAAAAGAT--	1	20
---AATTTGTAATATAAAAGATG---	1	20
--ATTTGTAATATAAAAGATG---	1	19
--GTAATATAAAAGATGAATTTGATTTG--	1	26
--GTAATATAAAAGATGAATTTG---	1	21
--AATATAAAAGATGAATTTGAT---	3	21
--**TCTTTTATATTATAAATTAGT**---	9	21

drnB-	Pooled			0h		16h	
	total	mir-5p	mir-3p	mir-5p	mir-3p	mir-5p	mir-3p
raw	10	7	0	1	0	6	0
rpm	0.1	0.1	0.0	0.03	0.0	0.11	0.0

ATATTCTTAACAATACATTTTATTTTAAAAAAAATGGTATTAATTTGTAATATAAAAGATGAATTTGATTTGTTTATTAGATTAATAATTAAATCAAATTCATCTTTTATATTATAAATTAGTACTATTTTTTTTTTTAAAAAAAAAAGTACAAGTTACAATAT	count	length
--**TAATTTGTAATATAAAAGATG**---	7	21
--GTAATATAAAAGATGAATTTG---	1	21
--AATATAAAAGATGAATTTGAT---	1	21
---TATATTATAAATTAGTACT--------------------------------------	1	19

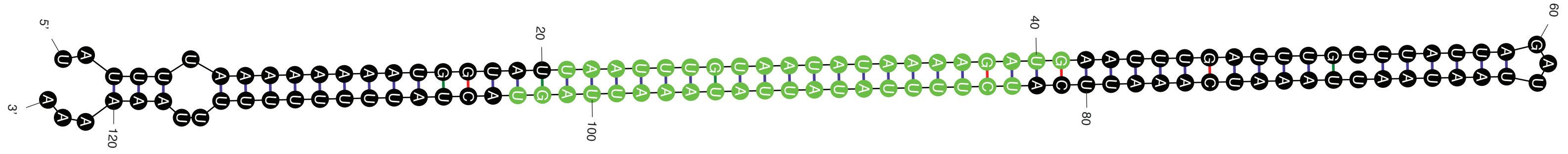

Supplementary Figure S2. Alignment of small RNA reads to each predicted miRNA hairpin including 20 bp flanking regions. Only perfect matches are included. For both wt and *drnB*$^-$ strains, the small RNA libraries from 0h and 16h developed cells are pooled. Mature miRNA sequences are indicated with bold text in the alignments and with green and blue in predicted hairpin structures. For each strain and miRNA, the pooled number of reads mapping to the precursor, pooled and time-point specific number of mature mir-5p and mir-3p (mature sequence +/- 1 nt at the 3' end) is given as both raw read counts and normalized to mapped library size (rpm).

miRNA: mir-1179

WT	Pooled			0h		16h	
	total	mir-5p	mir-3p	mir-5p	mir-3p	mir-5p	mir-3p
raw	99	39	47	0	0	39	47
rpm	1.0	0.4	0.5	0.0	0.0	0.76	0.89

TAAAATTACTTAAATCTATTTTTAATTCTTTGGGTCTCCATTCAAATTAAGTTCAGACTTAAAAAATTTCCTTAGTTTGAATTTTTTTTTAGGTATGAACTTAATTGGGTGGGACTAAAGAATTAAAACATTTGAGTAATTTTTTTTATTT count length
------------------------------TGGGTCTCCATTCAAATTAAGTT-- 1 23
------------------------------**TGGGTCTCCATTCAAATTAAGTTC**--- 38 24
-------------------------------------CCATTCAAATTAAGTTCAGACT-- 1 22
---TATGAACTTAATTGGGTGGGAC------------------------------------ 1 22
---TGAACTTAATTGGGTGGGACT----------------------------------- 1 21
--ACTTAATTGGGTGGGACT----------------------------------- 3 18
--**ACTTAATTGGGTGGGACTAAA**-------------------------------- 44 21
--ACTTAATTGGGTGGGACTA---------------------------------- 1 19
--ACTTAATTGGGTGGGACTAAAGAATTAAAAC---------------------- 3 31
--ACTTAATTGGGTGGGACTAA--------------------------------- 3 20
--ACTTAATTGGGTGGGACTAAAGAATTAAAACAT-------------------- 1 33
--TTAATTGGGTGGGACTAA--------------------------------- 1 18
---GGTGGGACTAAAGAATTAAAACATTT------------------ 1 26

drnB-	Pooled			0h		16h	
	total	mir-5p	mir-3p	mir-5p	mir-3p	mir-5p	mir-3p
raw	3	0	0	0	0	0	0
rpm	0.0	0.0	0.0	0.0	0.0	0.0	0.0

TAAAATTACTTAAATCTATTTTTAATTCTTTGGGTCTCCATTCAAATTAAGTTCAGACTTAAAAAATTTCCTTAGTTTGAATTTTTTTTTAGGTATGAACTTAATTGGGTGGGACTAAAGAATTAAAACATTTGAGTAATTTTTTTTATTT count length
---TGAACTTAATTGGGTGGGACT----------------------------------- 1 21
---AACTTAATTGGGTGGGACT----------------------------------- 1 19
--ACTTAATTGGGTGGGACTA---------------------------------- 1 19

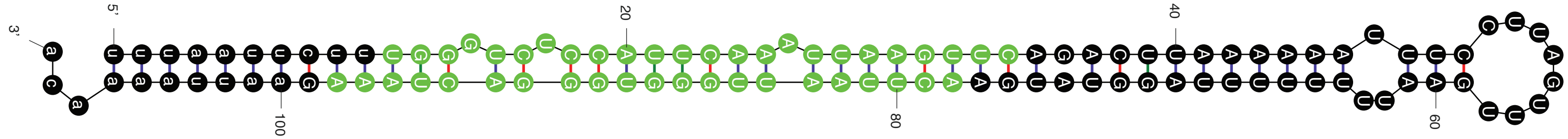

Supplementary Figure S2, continued.

miRNA: mir-1180-1; mir-1180-2

WT	Pooled			0h		16h		Pooled		0h		16h	
	total	mir-5p-1	mir-3p-1	mir-5p-1	mir-3p-1	mir-5p-1	mir-3p-1	mir-5p-2	mir-3p-2	mir-5p-2	mir-3p-2	mir-5p-2	mir-3p-2
raw	111	20	9	5	3	15	6	26	50	5	8	21	42
rpm	1.2	0.2	0.1	0.14	0.08	0.29	0.12	0.3	0.5	0.14	0.22	0.42	0.86

```
TTTTTACTTTTGAAACATTCTCTTCTCCAATCTTTTTTTCTGATGTGACATTTCAAATGACAAAGAATTTTTTATTTTTTTCTTTATTCTTTTTAATTAAAACTAAAGATTAATTAAAAAATTCTTATCTTTTGAAATGTGACTCAGCAAAAAAAGATTGGAGAAGAGAATGTTTAAACAGTAAAATGA count length
----------------------------AATCTTTTTTTCTGATGTGAC-------------------------------------------------------------------------------------------------------------------------------------------- 2 21
----------------------------AATCTTTTTTTCTGATGTGACA------------------------------------------------------------------------------------------------------------------------------------------- 18 22
--------------------------------------------------TTTCAAATGACAAAGAATTTTTT-------------------------------------------------------------------------------------------------------------------- 1 23
--------------------------------------------------TTTCAAATGACAAAGAATTTTT--------------------------------------------------------------------------------------------------------------------- 25 22
---------------------------------------------------------------------------------------------------------------------AAAATTCTTATCTTTTGAAATG-------------------------------------------------- 1 22
----------------------------------------------------------------------------------------------------------------------AAATTCTTATCTTTTGAAATG-------------------------------------------------- 49 21
----------------------------------------------------------------------------------------------------------------------AAATTCTTATCTTTTGAAAT--------------------------------------------------- 1 20
-----------------------------------------------------------------------------------------------------------------------AATTCTTATCTTTTGAAATG-------------------------------------------------- 1 20
------------------------------------------------------------------------------------------------------------------------ATTCTTATCTTTTGAAATG-------------------------------------------------- 1 19
-------------------------------------------------------------------------------------------------------------------------------------------TGACTCAGCAAAAAAAGAT------------------------------- 3 19
-------------------------------------------------------------------------------------------------------------------------------------------TGACTCAGCAAAAAAAGATTGGA--------------------------- 1 23
-------------------------------------------------------------------------------------------------------------------------------------------TGACTCAGCAAAAAAAGATTGG---------------------------- 8 22
```

drnB-	Pooled			0h		16h		Pooled		0h		16h	
	total	mir-5p-1	mir-3p-1	mir-5p-1	mir-3p-1	mir-5p-1	mir-3p-1	mir-5p-2	mir-3p-2	mir-5p-2	mir-3p-2	mir-5p-2	mir-3p-2
raw	1	0	0	0	0	0	0	0	0	0	0	0	0
rpm	0.0	0.0	0.0	0.0	0.0	0.0	0.0	0.0	0.0	0.0	0.0	0.0	0.0

```
TTTTTACTTTTGAAACATTCTCTTCTCCAATCTTTTTTTCTGATGTGACATTTCAAATGACAAAGAATTTTTTATTTTTTTCTTTATTCTTTTTAATTAAAACTAAAGATTAATTAAAAAATTCTTATCTTTTGAAATGTGACTCAGCAAAAAAAGATTGGAGAAGAGAATGTTTAAACAGTAAAATGA count length
--------------------------------------------------------------------------------------------------------------------------------------------------------AAAGATTGGAGAAGAGAATGT---------------- 1 21
```

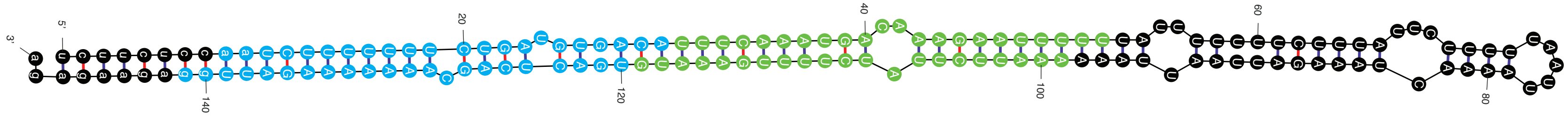

Supplementary Figure S2, continued.

miRNA: mir-1181

WT	Pooled			0h		16h	
	total	mir-5p	mir-3p	mir-5p	mir-3p	mir-5p	mir-3p
raw	867	842	2	280	1	562	1
rpm	9.9	9.5	0.0	7.65	0.03	11.29	0.02

```
AAAAAATAAGCACTAGATGACATACCCTTCTTTTTTAAATTGTGACCACTAGATACTACCTTCGAAAATAACGTACTTTCCAAAGTGATATCTAGCGATCACAATTTAAAAAAGAAGGTTATGTCATCTAGTGCTGCCATTGAGCT    count   length
--------AGCACTAGATGACATACCCTTC--------------------------------------------------------------------------------------------------------------------    4       22
----------CACTAGATGACATACCCTTC--------------------------------------------------------------------------------------------------------------------    1       20
------------CTAGATGACATACCCTTC--------------------------------------------------------------------------------------------------------------------    1       18
------------------------CCCTTCTTTTTTAAATTGTGACC---------------------------------------------------------------------------------------------------    1       23
------------------------CCCTTCTTTTTTAAATTGTGAC----------------------------------------------------------------------------------------------------    1       22
-------------------------CCTTCTTTTTTAAATTGTGAC----------------------------------------------------------------------------------------------------    1       21
---------------------------TTCTTTTTTAAATTGTGACC---------------------------------------------------------------------------------------------------    1       20
------------------------------TTTTTTAAATTGTGACCAC-------------------------------------------------------------------------------------------------    13      19
------------------------------TTTTTTAAATTGTGACCACT------------------------------------------------------------------------------------------------    733     20
------------------------------TTTTTTAAATTGTGACCACTA-----------------------------------------------------------------------------------------------    96      21
------------------------------TTTTTTAAATTGTGACCA--------------------------------------------------------------------------------------------------    2       18
-------------------------------TTTTTAAATTGTGACCACT------------------------------------------------------------------------------------------------    1       19
--------------------------------TTTTAAATTGTGACCACTAGA---------------------------------------------------------------------------------------------    7       21
------------------------------------AAATTGTGACCACTAGATACT-----------------------------------------------------------------------------------------    2       21
-----------------------------------------GTGACCACTAGATACTACCT-------------------------------------------------------------------------------------    1       20
----------------------------------------------------------------------------------------------GCGATCACAATTTAAAAAAGA-------------------------------    2       21
```

drnB-	Pooled			0h		16h	
	total	mir-5p	mir-3p	mir-5p	mir-3p	mir-5p	mir-3p
raw	404	115	1	36	1	79	0
rpm	4.4	1.2	0.0	0.83	0.02	1.49	0.0

```
AAAAAATAAGCACTAGATGACATACCCTTCTTTTTTAAATTGTGACCACTAGATACTACCTTCGAAAATAACGTACTTTCCAAAGTGATATCTAGCGATCACAATTTAAAAAAGAAGGTTATGTCATCTAGTGCTGCCATTGAGCT    count   length
------TAAGCACTAGATGACATACCC-----------------------------------------------------------------------------------------------------------------------    2       21
-------AAGCACTAGATGACATACCCT----------------------------------------------------------------------------------------------------------------------    1       21
--------AGCACTAGATGACATACCCTT---------------------------------------------------------------------------------------------------------------------    1       21
----------CACTAGATGACATACCCTTC--------------------------------------------------------------------------------------------------------------------    1       20
-----------ACTAGATGACATACCCTTCTTTTTTAAATTGTGAC----------------------------------------------------------------------------------------------------    1       35
-----------ACTAGATGACATACCCTTCT-------------------------------------------------------------------------------------------------------------------    1       20
---------------------ATACCCTTCTTTTTTAAATTGTGACCA--------------------------------------------------------------------------------------------------    1       27
---------------------ATACCCTTCTTTTTTAAATTG--------------------------------------------------------------------------------------------------------    1       21
----------------------TACCCTTCTTTTTTAAATTGTGACC---------------------------------------------------------------------------------------------------    1       25
----------------------TACCCTTCTTTTTTAAATTGT-------------------------------------------------------------------------------------------------------    3       21
----------------------TACCCTTCTTTTTTAAATTG--------------------------------------------------------------------------------------------------------    1       20
-------------------------CCTTCTTTTTTAAATTGTGAC----------------------------------------------------------------------------------------------------    3       21
-------------------------CCTTCTTTTTTAAATTGTGACCACT------------------------------------------------------------------------------------------------    1       25
-------------------------CCTTCTTTTTTAAATTGTGACC---------------------------------------------------------------------------------------------------    2       22
--------------------------CTTCTTTTTTAAATTGTGACCACT------------------------------------------------------------------------------------------------    1       24
--------------------------CTTCTTTTTTAAATTGTGACC---------------------------------------------------------------------------------------------------    11      21
---------------------------TTCTTTTTTAAATTGTGACCA--------------------------------------------------------------------------------------------------    1       21
---------------------------TTCTTTTTTAAATTGTGACCACT------------------------------------------------------------------------------------------------    1       23
---------------------------TTCTTTTTTAAATTGTGACC---------------------------------------------------------------------------------------------------    2       20
-----------------------------CTTTTTTAAATTGTGACCACT------------------------------------------------------------------------------------------------    1       21
------------------------------TTTTTTAAATTGTGACCACT------------------------------------------------------------------------------------------------    97      20
------------------------------TTTTTTAAATTGTGACCACTA-----------------------------------------------------------------------------------------------    18      21
------------------------------TTTTTTAAATTGTGACCA--------------------------------------------------------------------------------------------------    1       18
-------------------------------TTTTTAAATTGTGACCACTAG----------------------------------------------------------------------------------------------    2       21
-------------------------------TTTTTAAATTGTGACCACT------------------------------------------------------------------------------------------------    5       19
-------------------------------TTTTTAAATTGTGACCACTA-----------------------------------------------------------------------------------------------    1       20
--------------------------------TTTTAAATTGTGACCACTAGA---------------------------------------------------------------------------------------------    58      21
--------------------------------TTTTAAATTGTGACCACTAGAT--------------------------------------------------------------------------------------------    5       22
--------------------------------TTTTAAATTGTGACCACTAG----------------------------------------------------------------------------------------------    5       20
---------------------------------TTTAAATTGTGACCACTAGAT--------------------------------------------------------------------------------------------    19      21
```

Supplementary Figure S2, continued.

```
---------------------------------TTTAAATTGTGACCACTAGATA-------------------------------------------------------------------------------------------  1   22
---------------------------------TTTAAATTGTGACCACTAGA---------------------------------------------------------------------------------------------  1   20
----------------------------------TTAAATTGTGACCACTAGATA-------------------------------------------------------------------------------------------  2   21
----------------------------------TTAAATTGTGACCACTAGA---------------------------------------------------------------------------------------------  1   19
-----------------------------------TAAATTGTGACCACTAGATACT-----------------------------------------------------------------------------------------  1   22
-----------------------------------TAAATTGTGACCACTAGATAC------------------------------------------------------------------------------------------  6   21
------------------------------------AAATTGTGACCACTAGATACTACCTTCGAAAATAACGTACTTTCCAAAGTGA----------------------------------------------------------  1   52
------------------------------------AAATTGTGACCACTAGATACT-----------------------------------------------------------------------------------------  43  21
------------------------------------AAATTGTGACCACTAGATAC------------------------------------------------------------------------------------------  1   20
-------------------------------------AATTGTGACCACTAGATACTA----------------------------------------------------------------------------------------  5   21
-------------------------------------AATTGTGACCACTAGATACT-----------------------------------------------------------------------------------------  12  20
-------------------------------------AATTGTGACCACTAGATAC------------------------------------------------------------------------------------------  1   19
-------------------------------------AATTGTGACCACTAGATACTAC---------------------------------------------------------------------------------------  1   22
--------------------------------------ATTGTGACCACTAGATACTAC---------------------------------------------------------------------------------------  1   21
---------------------------------------TTGTGACCACTAGATACTACC--------------------------------------------------------------------------------------  2   21
---------------------------------------TTGTGACCACTAGATACTACCT-------------------------------------------------------------------------------------  2   22
----------------------------------------TGTGACCACTAGATACTACCT-------------------------------------------------------------------------------------  9   21
-----------------------------------------GTGACCACTAGATACTACCT-------------------------------------------------------------------------------------  1   20
-----------------------------------------GTGACCACTAGATACTACCTT------------------------------------------------------------------------------------  1   21
------------------------------------------TGACCACTAGATACTACCTTC-----------------------------------------------------------------------------------  3   21
--------------------------------------------ACCACTAGATACTACCTTCGA---------------------------------------------------------------------------------  1   21
-----------------------------------------------------TACTACCTTCGAAAATAACG-------------------------------------------------------------------------  1   20
--------------------------------------------------------TACCTTCGAAAATAACGTACT---------------------------------------------------------------------  4   21
---------------------------------------------------------ACCTTCGAAAATAACGTACT---------------------------------------------------------------------  1   20
------------------------------------------------------------------------------TCCAAAGTGATATCTAGCGAT-----------------------------------------------  1   21
-------------------------------------------------------------------------------CCAAAGTGATATCTAGCGATC----------------------------------------------  1   21
--------------------------------------------------------------------------------------------TAGCGATCACAATTTAAAAAA---------------------------------  4   21
--------------------------------------------------------------------------------------------TAGCGATCACAATTTAAAAAAG--------------------------------  1   22
---------------------------------------------------------------------------------------------AGCGATCACAATTTAAAAAAG--------------------------------  25  21
----------------------------------------------------------------------------------------------GCGATCACAATTTAAAAAAGA-------------------------------  1   21
-----------------------------------------------------------------------------------------------CGATCACAATTTAAAAAAGAA------------------------------  1   21
--------------------------------------------------------------------------------------------------TCACAATTTAAAAAAGAAGGT---------------------------  2   21
---------------------------------------------------------------------------------------------------CACAATTTAAAAAAGAAGGT---------------------------  1   20
-----------------------------------------------------------------------------------------------------------AAAAAAGAAGGTTATGTCAT-------------------  1   20
-----------------------------------------------------------------------------------------------------------AAAAAAGAAGGTTATGTCATC------------------  11  21
------------------------------------------------------------------------------------------------------------AAAAAGAAGGTTATGTCATC------------------  2   20
------------------------------------------------------------------------------------------------------------AAAAAGAAGGTTATGTCATCT-----------------  1   21
-------------------------------------------------------------------------------------------------------------------------TGTCATCTAGTGCTGCCATTG----  1   21
-------------------------------------------------------------------------------------------------------------------------TGTCATCTAGTGCTGCCATTGA---  1   22
```

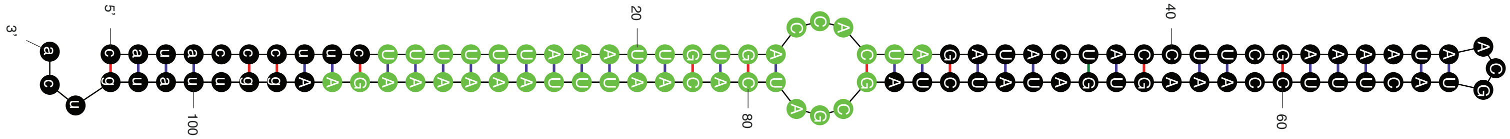

Supplementary Figure S2, continued.

miRNA: mir-1182

WT	Pooled			0h		16h	
	total	mir-5p	mir-3p	mir-5p	mir-3p	mir-5p	mir-3p
raw	226	174	31	30	5	144	26
rpm	2.5	1.9	0.3	0.82	0.14	2.97	0.51

ATATTCTTGGTAATGGTTATGTACCTGAGACACAGAATTTACCATTTGTGGTGCTATGGTTTAACAATAGGTCGAAATTTATTTGATTAAATAATGTGAAATTTTTCAAATAAATTTCGACCTCTCTTCAATCTGGCTCCCCAAATGATAAATTCTGTTTCTCAAGTATTTAACAATATCAAGATTATCAGA	count	length
--------GGTAATGGTTATGTACCTGAGA--	1	22
----------TAATGGTTATGTACCTGAGAC---	1	21
-----------------TATGTACCTGAGACACAGAATTTACCATTTGTGG---	1	34
-------------------------TGAGACACAGAATTTACCATT--	2	21
-----------------------------ACACAGAATTTACCATTTGTGG---	2	22
------------------------------CACAGAATTTACCATTTG--	4	18
------------------------------CACAGAATTTACCATTTGTGGT--	5	22
------------------------------**CACAGAATTTACCATTTGTGG**---	169	21
-------------------------------ACAGAATTTACCATTTGTGG---	3	20
---ACCTCTCTTCAATCTGGCTCCC---	1	22
---ACCTCTCTTCAATCTGGCTC---	2	20
---ACCTCTCTTCAATCTGGCTCC--	3	21
--**CCAAATGATAAATTCTGTTTC**-------------------------------	14	21
--CCAAATGATAAATTCTGTTTCT------------------------------	11	22
--CCAAATGATAAATTCTGTTT--------------------------------	6	20
--CCAAATGATAAATTCTGTT---------------------------------	1	19

drnB-	Pooled			0h		16h	
	total	mir-5p	mir-3p	mir-5p	mir-3p	mir-5p	mir-3p
raw	13	0	0	0	0	0	0
rpm	0.1	0.0	0.0	0.0	0.0	0.0	0.0

ATATTCTTGGTAATGGTTATGTACCTGAGACACAGAATTTACCATTTGTGGTGCTATGGTTTAACAATAGGTCGAAATTTATTTGATTAAATAATGTGAAATTTTTCAAATAAATTTCGACCTCTCTTCAATCTGGCTCCCCAAATGATAAATTCTGTTTCTCAAGTATTTAACAATATCAAGATTATCAGA	count	length
----------------TTATGTACCTGAGACACAGAAT--	1	22
----------------TTATGTACCTGAGACACAGAA---	6	21
------------------ATGTACCTGAGACACAGAATT---	1	21
-------------------TGTACCTGAGACACAGAATTT--	1	21
-------------------------TGAGACACAGAATTTACCA--	2	19
-------------------------TGAGACACAGAATTTACCATT--	1	21
--CCCCAAATGATAAATTCTGTT---------------------------------	1	21

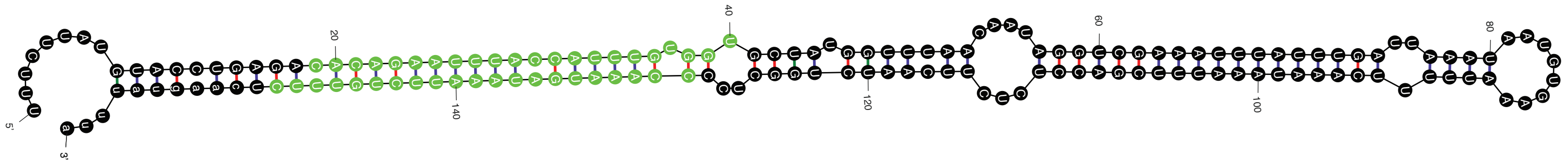

Supplementary Figure S2, continued.

miRNA: mir-1183

WT	Pooled			0h		16h	
	total	mir-5p	mir-3p	mir-5p	mir-3p	mir-5p	mir-3p
raw	2097	1840	62	53	2	1787	60
rpm	21.8	19.1	0.7	1.41	0.06	36.72	1.26

TCAGTGCCCTATAGCTGGTAGCAGACTTCCGTTTGCTTATACTATATAAGTGGTCGTTAATTGCTGGCAGACCTCAGTTTACTCACACTAGACCACTGGCCGTCTTCTGGCTGCTAGCGGGCAATGGCCTAGTATGAGTAAATGACCATCTGCCACCAGTTAGCGGTCATTTGTATAGCATGGGCAAACCCCAGTCTGCGAACATTAAGCTGACACTTA	count	length
TCAGTGCCCTATAGCTGGTAGCAGA--	2	25
TCAGTGCCCTATAGCTGGTAGCAGAC---	2	26
--AGTGCCCTATAGCTGGTAGCAGAC---	1	24
------CCCTATAGCTGGTAGCAGAC---	1	20
-------------------------CTTCCGTTTGCTTATACTATA---	1	21
--TATAAGTGGTCGTTAATTGCT--	2	21
--TAAGTGGTCGTTAATTGCTG---	8	20
--TAAGTGGTCGTTAATTGCT--	45	19
--**TAAGTGGTCGTTAATTGCTGGC**---	1814	22
--TAAGTGGTCGTTAATTGCTGGCA--	1	23
--TAAGTGGTCGTTAATTGCTGG--	25	21
--TAAGTGGTCGTTAATTGC---	25	18
---AAGTGGTCGTTAATTGCTGGC---	68	21
--AGTGGTCGTTAATTGCTG---	1	18
--AGTGGTCGTTAATTGCTGGC---	5	20
--AGTGGTCGTTAATTGCTGGCA--	1	21
--AATTGCTGGCAGACCTCAGTTTACTCACACT--	1	31
--TGGCAGACCTCAGTTTACTCACACT--	2	25
---CAGACCTCAGTTTACTCACAC---	1	21
--AGACCTCAGTTTACTCACACT--	13	21
---CTTCTGGCTGCTAGCGGGCAATGGCC--	1	26
--TATGAGTAAATGACCATCTGC--	1	21
---**CACCAGTTAGCGGTCATTTGTA**--	59	22
---CACCAGTTAGCGGTCATTTG--	1	20
---CACCAGTTAGCGGTCATTTGTATAGCATGGGC----------------------------------	2	32
---CACCAGTTAGCGGTCATTTGTAT---	1	23
---CACCAGTTAGCGGTCATTTGT---	2	21
---CACCAGTTAGCGGTCATT--	1	18
--ACCAGTTAGCGGTCATTTGTA--	1	21
---AGTTAGCGGTCATTTGTA--	1	18
---CGGTCATTTGTATAGCATGGG-----------------------------------	1	21
--ATAGCATGGGCAAACCCCAGT------------------------	1	21
---CTGCGAACATTAAGCTGACACTTA	5	24
--TGCGAACATTAAGCTGACACTTA	1	23

drnB-	Pooled			0h		16h	
	total	mir-5p	mir-3p	mir-5p	mir-3p	mir-5p	mir-3p
raw	1669	631	57	96	6	535	51
rpm	16.1	6.1	0.5	2.14	0.13	9.96	0.94

TCAGTGCCCTATAGCTGGTAGCAGACTTCCGTTTGCTTATACTATATAAGTGGTCGTTAATTGCTGGCAGACCTCAGTTTACTCACACTAGACCACTGGCCGTCTTCTGGCTGCTAGCGGGCAATGGCCTAGTATGAGTAAATGACCATCTGCCACCAGTTAGCGGTCATTTGTATAGCATGGGCAAACCCCAGTCTGCGAACATTAAGCTGACACTTA	count	length
-CAGTGCCCTATAGCTGGTAGCAGAC---	2	25
-CAGTGCCCTATAGCTGGTAGCAGACT--	2	26
--AGTGCCCTATAGCTGGTAGCAGAC---	27	24
--AGTGCCCTATAGCTGGTAGCAGACT--	8	25
---GTGCCCTATAGCTGGTAGCAGAC---	1	23
------CCCTATAGCTGGTAGCAGAC---	1	20
-------CCTATAGCTGGTAGCAGACT--	1	20
-------------------------CTTCCGTTTGCTTATACTATA---	2	21
-------------------------------------TATACTATATAAGTGGTCGTT---	3	21
---ATATAAGTGGTCGTTAATTGC---	6	21
---ATATAAGTGGTCGTTAATTGCT--	1	22
--TATAAGTGGTCGTTAATTGC---	12	20
--TATAAGTGGTCGTTAATTGCT--	159	21
--TATAAGTGGTCGTTAATTGCTG---	1	22
--TAAGTGGTCGTTAATTGCT--	80	19
--TAAGTGGTCGTTAATTGC---	20	18
--TAAGTGGTCGTTAATTGCTGGCAGACCTCAGTTTACTCACACT--	1	43
--TAAGTGGTCGTTAATTGCTGGCA--	6	23
--TAAGTGGTCGTTAATTGCTG---	27	20
--**TAAGTGGTCGTTAATTGCTGGC**---	590	22
--TAAGTGGTCGTTAATTGCTGG--	35	21
---AAGTGGTCGTTAATTGCTGGC---	326	21
--AGTGGTCGTTAATTGCTGGCA--	16	21
--AGTGGTCGTTAATTGCTGGC---	12	20
---GGTCGTTAATTGCTGGCAGAC---	1	21
---GTTAATTGCTGGCAGACCTCAGTTTACTCACACT--	1	34
---TAATTGCTGGCAGACCTCAGTTTACTCACACTAGACC---	1	37
--AATTGCTGGCAGACCTCAGTT--	4	21
--AATTGCTGGCAGACCTCAGTTTACTCACAC---	1	30
--AATTGCTGGCAGACCTCAGTTTACTCACA--	2	29
--AATTGCTGGCAGACCTCAGTTTACTCACACT--	4	31
---ATTGCTGGCAGACCTCAGTTTACTCACACT--	1	30
--TTGCTGGCAGACCTCAGTTTACTCACA--	1	27
--GCTGGCAGACCTCAGTTTACTCACACT--	1	27
---CTGGCAGACCTCAGTTTACTCACACT--	1	26
---CTGGCAGACCTCAGTTTACTCACAC---	1	25

Supplementary Figure S2, continued.

TGGCAGACCTCAGTTTACTC	3	20
TGGCAGACCTCAGTTTACTCACA	1	23
TGGCAGACCTCAGTTTACTCAC	12	22
TGGCAGACCTCAGTTTACTCACAC	2	24
TGGCAGACCTCAGTTTACTCACACT	5	25
GGCAGACCTCAGTTTACTCAC	3	21
GCAGACCTCAGTTTACTCAC	19	20
CAGACCTCAGTTTACTCACACT	1	22
CAGACCTCAGTTTACTCACAC	3	21
CAGACCTCAGTTTACTCAC	2	19
AGACCTCAGTTTACTCACAC	3	20
AGACCTCAGTTTACTCACA	1	19
AGACCTCAGTTTACTCACACT	4	21
TAGACCACTGGCCGTCTTCTGGCTGCT	1	27
TAGACCACTGGCCGTCTTCTGGCTGCTAGCGGGCAATGGCCTAGT	1	45
AGACCACTGGCCGTCTTCTGGC	1	22
GACCACTGGCCGTCTTCTGGC	4	21
CCACTGGCCGTCTTCTGGCTG	2	21
CACTGGCCGTCTTCTGGC	2	18
TCTTCTGGCTGCTAGCGGGCA	1	21
TTCTGGCTGCTAGCGGGC	1	18
TGGCCTAGTATGAGTAAATGAC	2	22
TATGAGTAAATGACCATCTGCCACCAGT	1	28
TATGAGTAAATGACCATCTGCC	2	22
TATGAGTAAATGACCATCTGC	5	21
TATGAGTAAATGACCATCTGCCACCAGTTAG	1	31
TATGAGTAAATGACCATCTG	1	20
ATGAGTAAATGACCATCTGCC	27	21
ATGAGTAAATGACCATCTGCCACC	6	24
ATGAGTAAATGACCATCTGC	3	20
TGAGTAAATGACCATCTGCC	2	20
GAGTAAATGACCATCTGCCACCAGTTAGCG	1	30
GAGTAAATGACCATCTGCCA	1	20
GAGTAAATGACCATCTGCCACCAGTT	1	26
TGACCATCTGCCACCAGTTAG	1	21
CATCTGCCACCAGTTAGCGGT	1	21
CACCAGTTAGCGGTCATTTG	3	20
**CACCAGTTAGCGGTCATTTGTA**	36	22
CACCAGTTAGCGGTCATTTGTATAGCATGGGC	1	32
CACCAGTTAGCGGTCATTTGT	21	21
ACCAGTTAGCGGTCATTTGT	1	20
ACCAGTTAGCGGTCATTTGTA	9	21
CCAGTTAGCGGTCATTTGTATA	1	22
CAGTTAGCGGTCATTTGTATA	3	21
AGTTAGCGGTCATTTGTATAG	1	21
AGTTAGCGGTCATTTGTATAGC	1	22
TTAGCGGTCATTTGTATAGC	11	20
TTAGCGGTCATTTGTATAGCA	6	21
AGCGGTCATTTGTATAGC	1	18
AGCGGTCATTTGTATAGCA	1	19
AGCGGTCATTTGTATAGCATG	1	21
CGGTCATTTGTATAGCATGGG	3	21
CGGTCATTTGTATAGCATGGGC	3	22
GGTCATTTGTATAGCATGGGC	1	21
TGTATAGCATGGGCAAACCCCAGTCTGCGAACATTAAGCTG	1	41
TATAGCATGGGCAAACCCCA	1	20
ATAGCATGGGCAAACCCCAG	1	20
TCTGCGAACATTAAGCTGACACTTA	4	25
CTGCGAACATTAAGCTGACACTT	1	23
CTGCGAACATTAAGCTGACACTTA	66	24
TGCGAACATTAAGCTGACACT	1	21
TGCGAACATTAAGCTGACACTTA	5	23

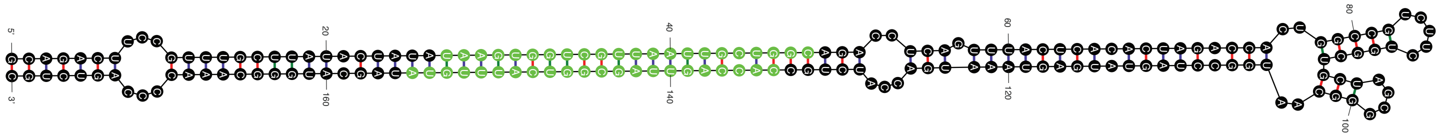

Supplementary Figure S2, continued.

miRNA: mir-1184

WT	Pooled			0h		16h	
	total	mir-5p	mir-3p	mir-5p	mir-3p	mir-5p	mir-3p
raw	581	17	438	14	252	3	186
rpm	7.1	0.2	5.3	0.37	6.81	0.06	3.84

```
GTATAATTTTAAATTTATATTATCAATCTACCAGTCATTTGGATCATCATCATCACTAAATGATGCACAAGAGTATTATGACTGTATTAGAGATGATGATGATGATCCAAATGACTGGTAGATTGATAATACTGTATTAGAGACT    count  length
----------------------------TACCAGTCATTTGGATCATCATC----------------------------------------------------------------------------------------------    1      23
------------------------------CCAGTCATTTGGATCATCATC----------------------------------------------------------------------------------------------    6      21
--------------------------------AGTCATTTGGATCATCATCATC-------------------------------------------------------------------------------------------    4      22
--------------------------------AGTCATTTGGATCATCATC----------------------------------------------------------------------------------------------    1      19
---------------------------------GTCATTTGGATCATCATC----------------------------------------------------------------------------------------------    3      18
---------------------------------GTCATTTGGATCATCATCATC-------------------------------------------------------------------------------------------    17     21
----------------------------------TCATTTGGATCATCATCATC-------------------------------------------------------------------------------------------    1      20
-----------------------------------------------------------------------------------------------ATGATGATGATCCAAATGACT-----------------------------    1      21
------------------------------------------------------------------------------------------------TGATGATGATCCAAATGACTGGT--------------------------    1      23
------------------------------------------------------------------------------------------------TGATGATGATCCAAATGACT-----------------------------    203    20
------------------------------------------------------------------------------------------------TGATGATGATCCAAATGAC------------------------------    18     19
------------------------------------------------------------------------------------------------TGATGATGATCCAAATGACTG----------------------------    232    21
------------------------------------------------------------------------------------------------TGATGATGATCCAAATGACTGG---------------------------    3      22
-------------------------------------------------------------------------------------------------GATGATGATCCAAATGACT-----------------------------    3      19
-------------------------------------------------------------------------------------------------GATGATGATCCAAATGACTGG---------------------------    2      21
-------------------------------------------------------------------------------------------------GATGATGATCCAAATGAC------------------------------    1      18
--------------------------------------------------------------------------------------------------ATGATGATCCAAATGACTGGT--------------------------    7      21
--------------------------------------------------------------------------------------------------ATGATGATCCAAATGACTGGTA-------------------------    1      22
---------------------------------------------------------------------------------------------------TGATGATCCAAATGACTGGTA-------------------------    69     21
---------------------------------------------------------------------------------------------------TGATGATCCAAATGACTGGT--------------------------    5      20
---------------------------------------------------------------------------------------------------TGATGATCCAAATGACTG----------------------------    1      18
-------------------------------------------------------------------------------------------------------GATCCAAATGACTGGTAGATT---------------------    1      21
```

Bold reads: **GTCATTTGGATCATCATCATC** (count 17), **TGATGATGATCCAAATGACTG** (count 232).

drnB-	Pooled			0h		16h	
	total	mir-5p	mir-3p	mir-5p	mir-3p	mir-5p	mir-3p
raw	5	0	0	0	0	0	0
rpm	0.1	0.0	0.0	0.0	0.0	0.0	0.0

```
GTATAATTTTAAATTTATATTATCAATCTACCAGTCATTTGGATCATCATCATCACTAAATGATGCACAAGAGTATTATGACTGTATTAGAGATGATGATGATGATCCAAATGACTGGTAGATTGATAATACTGTATTAGAGACT    count  length
----------------------TCAATCTACCAGTCATTTGGAT-----------------------------------------------------------------------------------------------------    1      22
-----------------------CAATCTACCAGTCATTTGGAT-----------------------------------------------------------------------------------------------------    1      21
--------------------------------------------------------------------------------------TTAGAGATGATGATGATGATC--------------------------------------    3      21
```

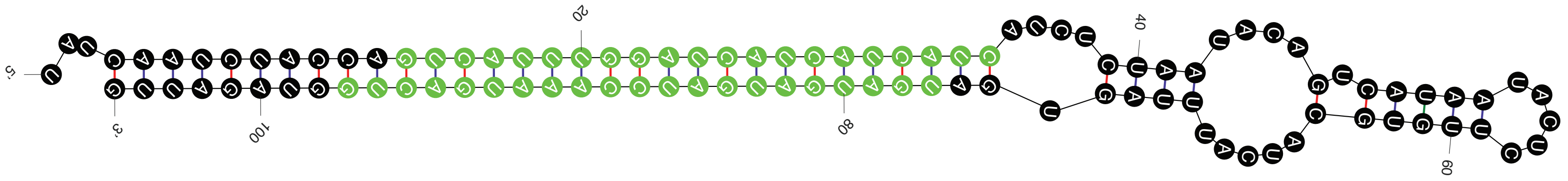

Supplementary Figure S2, continued.

miRNA: mir-1185

WT	Pooled			0h		16h	
	total	mir-5p	mir-3p	mir-5p	mir-3p	mir-5p	mir-3p
raw	1017	41	880	37	488	10	392
rpm	12.2	0.6	10.4	1.03	13.57	0.19	7.32

```
AGTGGATAAATCTCCAATGAGTATTATCGTTAATGTATTCTTGATATCTATTATAACCAATTTCCTCCAATTTATATTTACTTGTGATCAATACCAATTGTAGTAAATATAAATTGGAGGAAATTGGTTATATTAGATATCAAGAATACATCAACGATAATACTGTTATAGTCTCCCATCAAT count length
---------ATCTCCAATGAGTATTATCGT--------------------------------------------------------------------------------------------------------------------------------------------------------- 2 21
--------------------------TCGTTAATGTATTCTTGATAT---------------------------------------------------------------------------------------------------------------------------------------- 5 21
--------------------------TCGTTAATGTATTCTTGATA----------------------------------------------------------------------------------------------------------------------------------------- 3 20
---------------------------CGTTAATGTATTCTTGATATC--------------------------------------------------------------------------------------------------------------------------------------- 47 21
----------------------------------------------TCTATTATAACCAATTTCCTCC------------------------------------------------------------------------------------------------------------------- 24 22
----------------------------------------------TCTATTATAACCAATTTCCTC-------------------------------------------------------------------------------------------------------------------- 5 21
-----------------------------------------------CTATTATAACCAATTTCCTCC------------------------------------------------------------------------------------------------------------------- 8 21
------------------------------------------------TATTATAACCAATTTCCTCCA------------------------------------------------------------------------------------------------------------------ 6 21
------------------------------------------------TATTATAACCAATTTCCTCC------------------------------------------------------------------------------------------------------------------- 5 20
------------------------------------------------TATTATAACCAATTTCCTCCAAT---------------------------------------------------------------------------------------------------------------- 2 23
---------------------------------------------------TATAACCAATTTCCTCCAATT--------------------------------------------------------------------------------------------------------------- 1 21
--------------------------------------------------------------------------TATTTACTTGTGATCAATACC---------------------------------------------------------------------------------------- 1 21
--------------------------------------------------------------------------------------------------------------------GAGGAAATTGGTTATATTAGA---------------------------------------------- 3 21
---------------------------------------------------------------------------------------------------------------------AGGAAATTGGTTATATTAGATA-------------------------------------------- 1 22
---------------------------------------------------------------------------------------------------------------------AGGAAATTGGTTATATTAGAT--------------------------------------------- 1 21
----------------------------------------------------------------------------------------------------------------------GGAAATTGGTTATATTAGATA-------------------------------------------- 2 21
------------------------------------------------------------------------------------------------------------------------AAATTGGTTATATTAGATATC------------------------------------------ 2 21
-------------------------------------------------------------------------------------------------------------------------AATTGGTTATATTAGATATC------------------------------------------ 2 20
-----------------------------------------------------------------------------------------------------------------------------------------TATCAAGAATACATCAACGAT------------------------- 880 21
------------------------------------------------------------------------------------------------------------------------------------------ATCAAGAATACATCAACGAT------------------------- 9 20
------------------------------------------------------------------------------------------------------------------------------------------ATCAAGAATACATCAACGATA------------------------ 2 21
-------------------------------------------------------------------------------------------------------------------------------------------TCAAGAATACATCAACGAT------------------------- 2 19
-------------------------------------------------------------------------------------------------------------------------------------------TCAAGAATACATCAACGATAA----------------------- 4 21
```

drnB-	Pooled			0h		16h	
	total	mir-5p	mir-3p	mir-5p	mir-3p	mir-5p	mir-3p
raw	82	0	6	0	2	0	8
rpm	1.0	0.0	0.1	0	0.05	0	0.08

```
AGTGGATAAATCTCCAATGAGTATTATCGTTAATGTATTCTTGATATCTATTATAACCAATTTCCTCCAATTTATATTTACTTGTGATCAATACCAATTGTAGTAAATATAAATTGGAGGAAATTGGTTATATTAGATATCAAGAATACATCAACGATAATACTGTTATAGTCTCCCATCAAT count length
----------TCTCCAATGAGTATTATCGTT-------------------------------------------------------------------------------------------------------------------------------------------------------- 1 21
---------------------TATTATCGTTAATGTATTCT---------------------------------------------------------------------------------------------------------------------------------------------- 1 20
------------------------TATCGTTAATGTATTCTTGATATCTATTATAACCAATTTCC---------------------------------------------------------------------------------------------------------------------- 1 41
-------------------------------AATGTATTCTTGATATCTATT----------------------------------------------------------------------------------------------------------------------------------- 1 21
-------------------------------------TTCTTGATATCTATTATAACC----------------------------------------------------------------------------------------------------------------------------- 1 21
----------------------------------------------TCTATTATAACCAATTTCCTCC------------------------------------------------------------------------------------------------------------------- 2 22
----------------------------------------------TCTATTATAACCAATTTCCTC-------------------------------------------------------------------------------------------------------------------- 2 21
-----------------------------------------------CTATTATAACCAATTTCCTCC------------------------------------------------------------------------------------------------------------------- 2 21
--------------------------------------------------TTATAACCAATTTCCTCCAAT---------------------------------------------------------------------------------------------------------------- 1 21
----------------------------------------------------ATAACCAATTTCCTCCAATTT-------------------------------------------------------------------------------------------------------------- 2 21
-----------------------------------------------------TAACCAATTTCCTCCAATTT-------------------------------------------------------------------------------------------------------------- 1 20
----------------------------------------------------------AATTTCCTCCAATTTATATTTACTTGTGATCAATACCAATTGTAGT------------------------------------------------------------------------------- 1 46
-----------------------------------------------------------ATTTCCTCCAATTTATATTTA------------------------------------------------------------------------------------------------------- 2 21
---------------------------------------------------------------------------ATTTACTTGTGATCAATACC---------------------------------------------------------------------------------------- 2 20
----------------------------------------------------------------------------------------------CAATTGTAGTAAATATAAATTGGA----------------------------------------------------------------- 1 24
--------------------------------------------------------------------------------------------------------AAATATAAATTGGAGGAAAT----------------------------------------------------------- 1 20
---------------------------------------------------------------------------------------------------------AATATAAATTGGAGGAAATT---------------------------------------------------------- 3 20
-----------------------------------------------------------------------------------------------------------TATAAATTGGAGGAAATTGGT------------------------------------------------------- 1 21
---------------------------------------------------------------------------------------------------------------AATTGGAGGAAATTGGTTATA--------------------------------------------------- 1 21
------------------------------------------------------------------------------------------------------------------------AAATTGGTTATATTAGATATC------------------------------------------ 26 21
-------------------------------------------------------------------------------------------------------------------------AATTGGTTATATTAGATATC------------------------------------------ 16 20
------------------------------------------------------------------------------------------------------------------------------------TTAGATATCAAGAATACATC------------------------------- 1 20
-----------------------------------------------------------------------------------------------------------------------------------------TATCAAGAATACATCAACGAT------------------------- 6 21
------------------------------------------------------------------------------------------------------------------------------------------ATCAAGAATACATCAACGAT------------------------- 2 20
-------------------------------------------------------------------------------------------------------------------------------------------TCAAGAATACATCAACGATAAT---------------------- 1 22
-------------------------------------------------------------------------------------------------------------------------------------------TCAAGAATACATCAACGATAA----------------------- 2 21
--------------------------------------------------------------------------------------------------------------------------------------------------TACATCAACGATAATACTGT----------------- 1 20
```

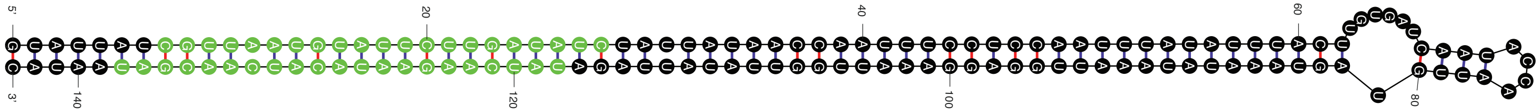

Supplementary Figure S2, continued.

miRNA: mir-7102-1; mir-7102-2

WT	Pooled			0h		16h		Pooled		0h		16h	
	total	mir-5p-1	mir-3p-1	mir-5p-1	mir-3p-1	mir-5p-1	mir-3p-1	mir-5p-2	mir-3p-2	mir-5p-2	mir-3p-2	mir-5p-2	mir-3p-2
raw	2795	426	10	148	6	278	4	808	1171	316	436	492	735
rpm	32.3	4.8	0.1	4.01	0.17	5.63	0.08	9.4	13.4	8.6	11.89	10.23	14.98

ATTTAATTCATCTTTATTATTATTGTTGAACTCTTGTCTTAATTCCTTGATAGCTTTATAGCTCGTAGCATTTTCTTCGTCATTTGTTTATTGGATATTCCTAAAGATGAATTATCAAGAAAATGTTACGAACTATAAATCTATCAAAGAAGTAAGATAAGAGTTCAACAATCTCATTAGAGATGAATTAAAT	count	length
-----------CTTTATTATTATTGTTGAACT---	1	21
------------TTTATTATTATTGTTGAACTCT---	1	22
------------------ATTATTGTTGAACTCTTGTCT--	1	21
-------------------------TTGAACTCTTGTCTTAATTCCT--	2	22
-------------------------------TCTTGTCTTAATTCCTTGATAGC---	6	23
-------------------------------TCTTGTCTTAATTCCTTGATAG--	3	22
--------------------------------CTTGTCTTAATTCCTTGATAG--	3	21
---------------------------------TTGTCTTAATTCCTTGATAGCT--	171	22
---------------------------------TTGTCTTAATTCCTTGATAGCTT---	5	23
---------------------------------**TTGTCTTAATTCCTTGATAGC**---	246	21
---------------------------------TTGTCTTAATTCCTTGATAG--	9	20
----------------------------------TGTCTTAATTCCTTGATAGCTT---	3	22
----------------------------------TGTCTTAATTCCTTGATAGC---	10	20
----------------------------------TGTCTTAATTCCTTGATAGCT--	165	21
-------------------------------------CTTAATTCCTTGATAGCT--	1	18
---CTTTATAGCTCGTAGCATTT--	1	20
---CTTTATAGCTCGTAGCATTTTC--	18	22
---CTTTATAGCTCGTAGCATTTT---	1	21
---CTTTATAGCTCGTAGCATTTTCT---	1	23
---CTTTATAGCTCGTAGCATTTTCTT--	1	24
--TTTATAGCTCGTAGCATTTTCT---	20	22
--TTTATAGCTCGTAGCATTTT---	7	20
--TTTATAGCTCGTAGCATTTTC--	37	21
--TTTATAGCTCGTAGCATTT--	1	19
---TTATAGCTCGTAGCATTTTCTT--	8	22
---TTATAGCTCGTAGCATTTTC--	53	20
---**TTATAGCTCGTAGCATTTTCT**---	755	21
--TATAGCTCGTAGCATTTTCTT--	1	21
--TATAGCTCGTAGCATTTTCTTC---	2	22
--TATAGCTCGTAGCATTTTCT---	5	20
--TATAGCTCGTAGCATTTTC--	1	19
--TCAAGAAAATGTTACGAACT---	1	20
---AAAATGTTACGAACTATAAAT---	33	21
---AAAATGTTACGAACTATAAATCT---	1	23
---AAAATGTTACGAACTATAAATC--	5	22
---AAAATGTTACGAACTATAAA--	1	20
--AAATGTTACGAACTATAAAT---	49	20
--AAATGTTACGAACTATAAA--	3	19
--AAATGTTACGAACTATAAATCT---	137	22
--**AAATGTTACGAACTATAAATC**--	985	21
---AATGTTACGAACTATAAATC--	2	20
---AATGTTACGAACTATAAATCT---	1	21
--CTATCAAAGAAGTAAGATAAG--------------------------------	11	21
--CTATCAAAGAAGTAAGATAAGA-------------------------------	6	22
---TATCAAAGAAGTAAGATAAG--------------------------------	9	20
---**TATCAAAGAAGTAAGATAAGA**-------------------------------	1	21
--ATCAAAGAAGTAAGATAAGAG------------------------------	1	21
--ATCAAAGAAGTAAGATAAGAGT-----------------------------	10	22

drnB-	Pooled			0h		16h		Pooled		0h		16h	
	total	mir-5p-1	mir-3p-1	mir-5p-1	mir-3p-1	mir-5p-1	mir-3p-1	mir-5p-2	mir-3p-2	mir-5p-2	mir-3p-2	mir-5p-2	mir-3p-2
raw	29	4	0	1	0	3	0	2	14	0	2	2	12
rpm	0.3	0.0	0.0	0.02	0.0	0.6	0.0	0.0	0.1	0.0	0.04	0.04	0.23

ATTTAATTCATCTTTATTATTATTGTTGAACTCTTGTCTTAATTCCTTGATAGCTTTATAGCTCGTAGCATTTTCTTCGTCATTTGTTTATTGGATATTCCTAAAGATGAATTATCAAGAAAATGTTACGAACTATAAATCTATCAAAGAAGTAAGATAAGAGTTCAACAATCTCATTAGAGATGAATTAAAT	count	length
-------------------------TTGAACTCTTGTCTTAATTCC---	1	21
--------------------------TGAACTCTTGTCTTAATTCCT--	7	21
---------------------------------**TTGTCTTAATTCCTTGATAGC**---	4	21
---**TTATAGCTCGTAGCATTTTCT**---	2	21
--**AAATGTTACGAACTATAAATC**--	14	21
---AAAGAAGTAAGATAAGAGTTC---------------------------	1	21

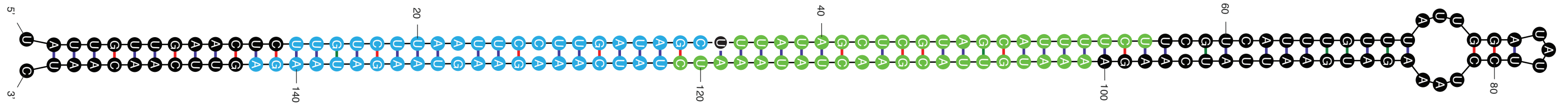

Supplementary Figure S2, continued.

A. U6 control for semi-qRT-PCR for pri-mir-1176

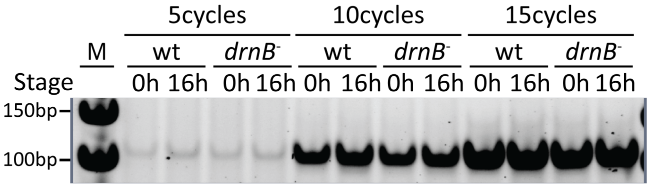

B. U6 control for semi-qRT-PCR for pri-mir-1177

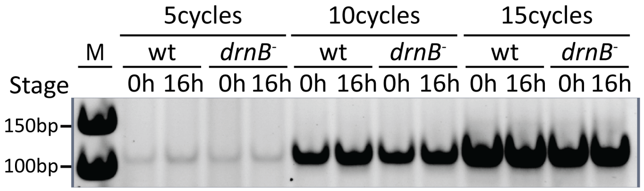

Supplementary Figure S3. (A,B) RT-PCR analysis of U6 spliceosomal RNA expression to determine the optimal number of PCR cycles for semi-qRT-PCR of pri-mir-1176 and 1177. RNA was isolated from wt and DrnB depleted (*drnB*$^-$) cells during growth (0h) and development (16h). cDNA templates were synthesized using oligonucleotides complementary to U6 (and pri-mir-1176 (A) or pri-mir-1177 (B)).

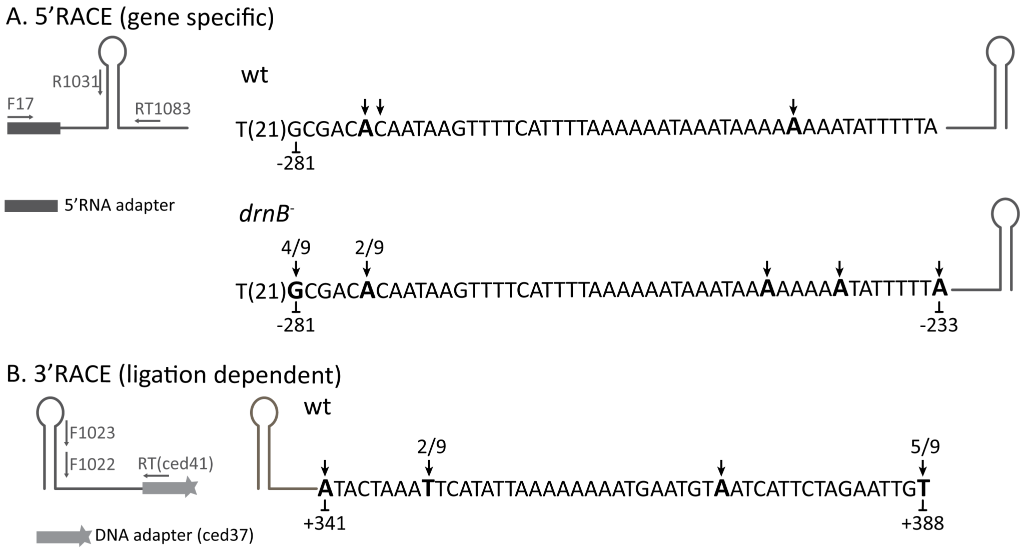

Supplementary Figure S4. Pri-mir-1176 5´ and 3' ends. A and B show all additional 5´ and 3´ ends, respectively, indicated in figure 3 A and C, as vertical arrows above the sequences. 5' and 3' nucleotides from multiple RACE clones are indicated by numbers as fraction/total sequenced RACE-clones. Numbers below the sequences indicate distance to the 5'-end of miRNA-5p. Arrows in the schematic pri-mir-1176 figures represent primers used for the analyses where prefixes RT, F, and R stand for reverse transcription, forward, and reverse, respectively. The 5´ and 3´ adapters ligated to the RNA are indicated as filled boxes.

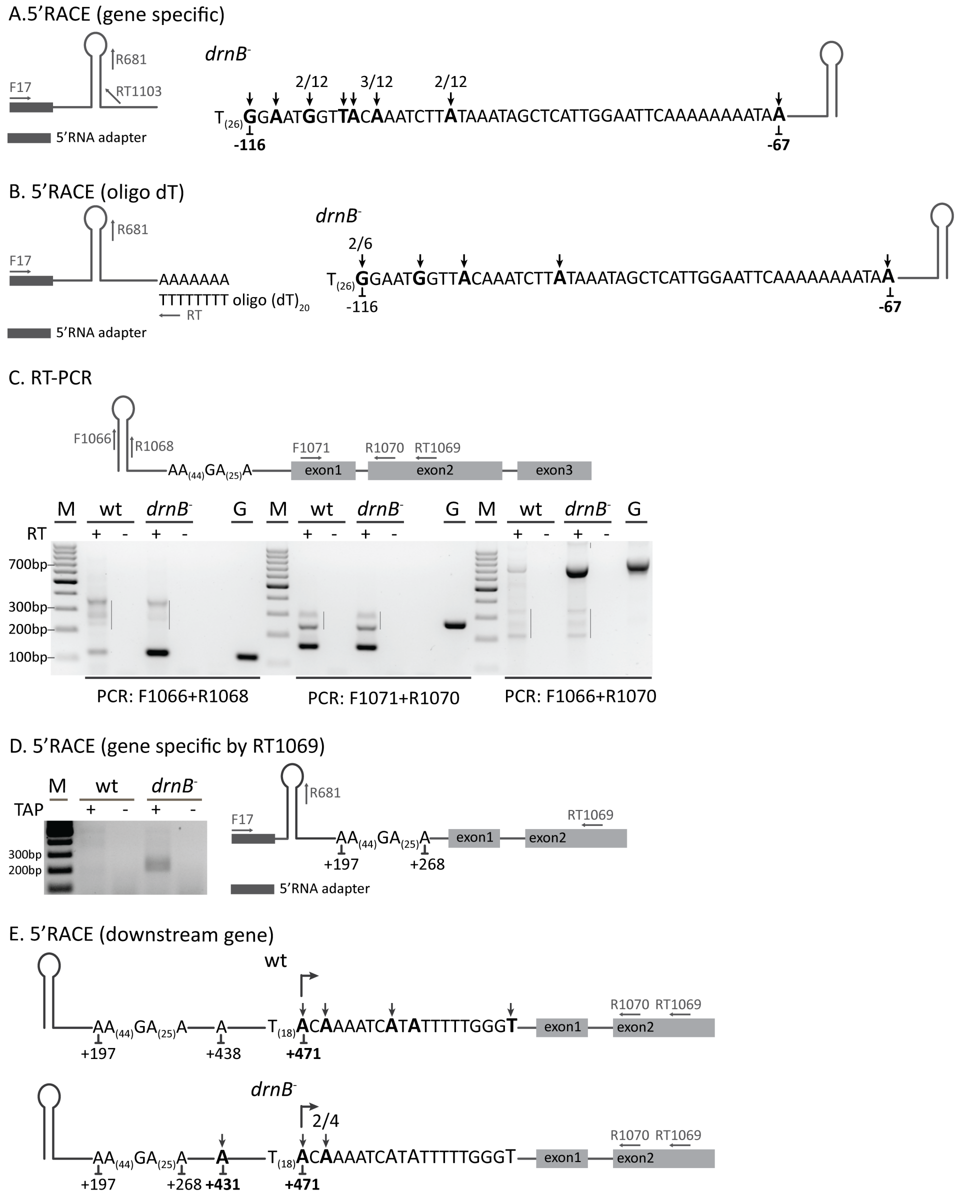

A.5'RACE (gene specific)
R681
F17
RT1103
5'RNA adapter
drnB-
2/12
3/12
2/12
T(26)GGAATGGTTACAAATCTTATAAATAGCTCATTGGAATTCAAAAAAAAATAA
-116
-67
B. 5'RACE (oligo dT)
R681
F17
AAAAAAA
TTTTTTTT oligo (dT)20
RT
5'RNA adapter
drnB-
2/6
T(26)GGAATGGTTACAAATCTTATAAATAGCTCATTGGAATTCAAAAAAAAATAA
-116
-67
C. RT-PCR
F1066
R1068
F1071
R1070
RT1069
AA(44)GA(25)A
exon1
exon2
exon3
M
wt
drnB-
G
RT
+
-
700bp
300bp
200bp
100bp
PCR: F1066+R1068
PCR: F1071+R1070
PCR: F1066+R1070
D. 5'RACE (gene specific by RT1069)
M
wt
drnB-
TAP
300bp
200bp
R681
F17
RT1069
AA(44)GA(25)A
exon1
exon2
+197
+268
5'RNA adapter
E. 5'RACE (downstream gene)
wt
R1070
RT1069
AA(44)GA(25)A—A—T(18)ACAAAATCATATTTTTGGGT
exon1
exon2
+197
+438
+471
drnB-
2/4
R1070
RT1069
AA(44)GA(25)A—A—T(18)ACAAAATCATATTTTTGGGT
exon1
exon2
+197
+268
+431
+471

Supplementary Figure S5. Transcription and 5’-ends of pri-mir-1177 and the downstream gene. The primer prefixes RT, F, and R stand for primers used for reverse transcription, forward, and revers PCR primers, respectively. Numbers below sequences indicate the distance (nucleotides) from the start of miRNA-5p. Vertical arrows denote determined 5’-ends (A,B,E,). (A,B) correspond to figures 5A and B, respectively, and display additional 5’-ends of pri-mir-1177 from cloned 5’RACE products where reverse transcriptions were initiated with locus specific (A) and oligo (dT) (B) primers. The RNA oligonucleotide ligated to the 5’-ends of the RNA is shown as a filled box. (C) RT-PCR analysis of transcripts from the miRNA locus and the downstream gene. Results from the agarose gel electrophoresis are shown where primers used are specified below and their genomic positions indicated in the schematic structure on top. G shows the genomic PCR controls and vertical lines mark unspecific PCR products. (D) 5´RACE products analyzed by agarose gel electrophoresis. The position of the primer used for reverse transcription is shown above exon 2. +/- TAP denotes RNA samples treated or untreated, respectively, with Tobacco Acid Pyrophosphatase (TAP) prior to ligation of the RNA oligo. (E) 5’RACE of the downstream gene: correspond to figure 5D and show additional 5’-ends of transcripts from the downstream protein coding gene. Bent arrows indicate the suggested TSS.

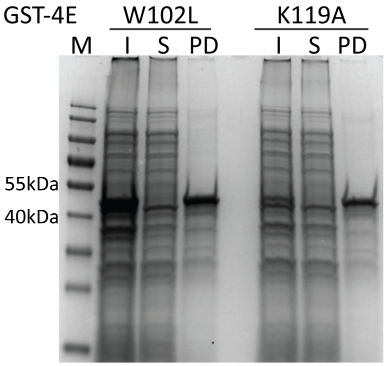

Supplementary Figure S6. Purification of GST-4E proteins. GST tagged variants were expressed in *Escherichia coli* and lysates were incubated with glutathione magnetic beads. After washing and releasing the GST-4E proteins from the beads, aliquots from input (I), supernatant (S) and pulled down (PD) fractions were analyzed by polyacrylamide gel electrophoresis.

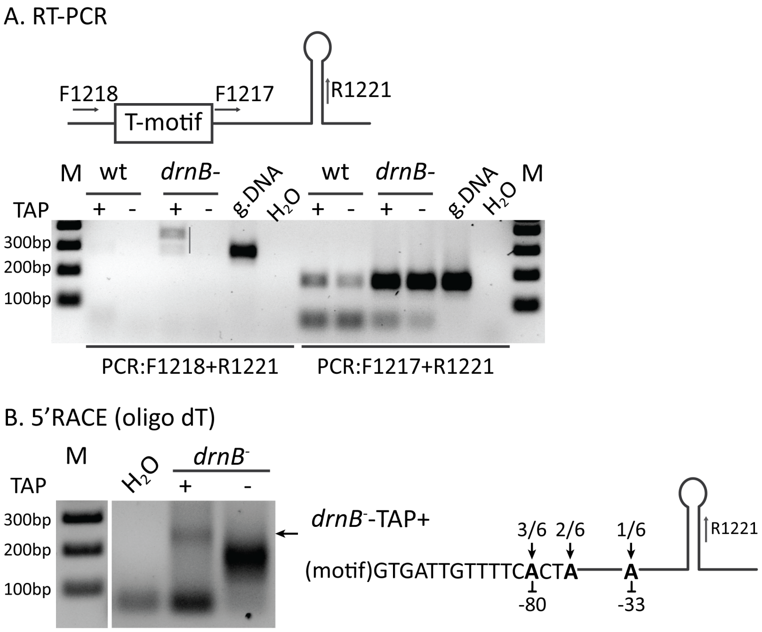

Supplementary Figure S7. Pri-mir-7097 transcription starts downstream of the conserved T-rich motif. Arrows in the schematic pri-mir-7097 figures represent primers used for the analyses where prefixes F and R stand for forward and reverse, respectively. Tobacco Acid Pyrophosphatase (TAP) was used to remove cap-structures before an RNA-oligonucleotide was ligated to the 5´-ends whereafter an oligo (dT) primer was used for reverse transcription (see Material and Methods). (A) RT-PCR to confirm the presence of transcripts starting downstream of the conserved T-rich motif (T-motif). Genomic DNA (g.DNA) template was used as positive control. Vertical bar indicates unspecific PCR products. (B) 5´-ends determined by RACE experiments. 5´-ends (horizontal arrows) from multiple RACE clones were sequenced and are indicated by numbers as fraction/total sequenced RACE-clones. Numbers below the sequences denote nucleotide positions in relation to the 5´ most nucleotide of mir-5p. The RACE products from TAP- sample are most likely due to unspecific primer binding and/or amplified degradation products.

Supplementary Table S1. Identified DrnB-dependent miRNA candidates. Each candidate was evaluated based on the high confidence criteria proposed by Kozomara, A., & Griffiths-Jones, S. (2014): 1) mature mir-5p and mir-3p should be represented by at least 10 sequencing reads with no mismatches, 2) most abundant read pair should pair with 0-4 nt 3' overhang, 3) At least 50 % of the reads should have the same 5' end on each half of the hairpin, 4) the predicted hairpin should have a folding free energy of less than -0.2 kcal/mol/nt, 5) At least 60 % of the bases in the mature miRNA duplex should pair. Pooled small RNA sequencing data from this study and data from AX2 wt cells from Avesson et al., 2012 were used to evaluate each candidate. miRNA_can_D1 and miRNA_can_D2 were identified in Meier et al., 2016.

						High confidence criteria						
						1		2	3		4	5
miRNA	Locus	Strand	mir-5p	mir-3p	Genomic context	mir-5p	mir-3p		5' half	3' half		
mir-1178	DDB0232429:580102-580224	+	TAATTTGTAATATAAAAGATG	TCTTTTATATTATAAATTAGT	Intergenic	141	9	yes	95%	100%	-0.58	yes
mir-1179	DDB0232429:1649209-1649317	-	TGGGTCTCCATTCAAATTAAGTTC	ACTTAATTGGGTGGGACTAAA	Intergenic	121	71	yes	95%	92%	-0.52	yes
mir-1180-1	DDB0232433:2518294-2518442	-	AATCTTTTTTTCTGATGTGACA	TGACTCAGCAAAAAAAGATTGG	Intron of DDB_G0293106	22	14	yes	100%	95%	-0.53	yes
mir-1180-2	DDB0232433:2518294-2518442	-	TTTCAAATGACAAAGAATTTTT	AAATTCTTATCTTTTGAAATG	(sense)	26	53	yes	100%	95%	-0.53	yes
mir-1181	DDB0232428:2717082-2717187	-	TTTTTTAAATTGTGACCACT	GCGATCACAATTTAAAAAAGA	Intergenic	842	17	yes	98%	95%	-0.52	yes
mir-1182	DDB0232430:3663221-3663372	-	CACAGAATTTACCATTTGTGG	CCAAATGATAAATTCTGTTTC	Intron of DDB_G0280709 (antisense)	175	32	yes	96%	83%	-0.52	yes
mir-1183	DDB0232431:735791-735969	-	TAAGTGGTCGTTAATTGCTGGC	CACCAGTTAGCGGTCATTTGTA	Intergenic	1840	62	yes	95%	89%	-0.58	yes
mir-1184	DDB0232431:1300154-1300258	+	GTCATTTGGATCATCATCATC	TGATGATGATCCAAATGACTG	Exon of DDB_G0283997 (pseudogene)	18	454	yes	62%	83%	-0.72	yes
mir-1185	DDB0232433:958097-958239	-	CGTTAATGTATTCTTGATATC	TATCAAGAATACATCAACGAT	Intergenic	48	882	yes	39%	95%	-0.73	yes
mir-7102-2	DDB0232431:2377503-2377655	-	TTATAGCTCGTAGCATTTTCT	AAATGTTACGAACTATAAATC	Intron of DDB_G0284713	816	1176	yes	80%	95%	-0.62	yes
miRNA_can_D1	DDB0232431:1706344-1706488	+	TCTTTCTCTAATTTCATTTATT	AAATGAAATTAGAGAAAGGGAT	Intergenic	209	330	yes	62%	97%	-0.69	yes
miRNA_can_D2	DDB0232432:4871883-4872029	+	TTCTCGACAGACATAGCATTGG	AATGCTTAGATGTAGAGGAAAC	Intergenic	55	10	yes	88%	56%	-0.51	yes

Supplementary Table S2. Oligonucleotides. Olgonucleotides used for reverse transcription (RT) and forward (F) and reverse (R) primers for PCR. Column "Experiments": The experiment where the primers were used were GS, LD, and DSG indicate gene specific, ligation

Oligo name	Target/description	Experiments	Sequence (5' to 3')
RT961	mir-1177	Stem-loop RT-qPCR	GTCGTATCCAGTGCAGGGTCCGAGGTATTCGCACTGGATACGACGAACCA
RT963	mir-1176	Stem-loop RT-qPCR	GTCGTATCCAGTGCAGGGTCCGAGGTATTCGCACTGGATACGACGCTTTC
F962	mir-1177	Stem-loop RT-qPCR	CGCGCCCAGTTAGGGTTTAA
F964	mir-1176	Stem-loop RT-qPCR	CGCGCCCAATTTTTATCAAG
R969	Reverse primer	Stem-loop RT-qPCR	GTGCAGGGTCCGAGGT
RT103	snRNA U6	Stem-loop RT-qPCR<br>Semi-qRT-PCR<br>Cap pull-down RT-PCR	CTTTACGGTCATCCTTACGCAGGGA
R20	snRNA U6	Stem-loop RT-qPCR	CGGTCATCCTTACGCAGGGACCAT
F104	snRNA U6	Semi-RT-qPCR,<br>Cap pull-down RT-PCR	GGGCAACCGGCAGGCATCCA
102	snRNA U6	Northern blot	GGATGCCTGCCGGTTGCCCGGAGG
1121	mir-1185-3p	Northern blot	ATCGTTGATGTATTCTTGATA
885	mir-1183-5p	Northern blot	GCCAGCAATTAACGACCACTT
GeneRacer RNA oligo	5'RNA adaptor	5'RACE (RT)	CGACUGGAGCACGAGGACACUGACAUGGACUGAAGGAGUAGAAA
F17	GeneRacer 5' primer	5'RACE	CGACTGGAGCACGAGGACACTGA
RT1083	pri-mir-1176	Semi-qRT-PCR<br>5'RACE (GS)-RT<br>Cap pull-down RT-PCR	TTCACTCGATCAGAGAAGAATC
R1031	pri-mir-1176	Semi-qRT-PCR<br>5'RACE<br>PCR verification of TSS<br>Cap pull-down RT-PCR	GCTTTCCTTGATAAAAATTGGCCA
F1026	pri-mir-1176	Semi-qRT-PCR<br>PCR verification of TSS<br>Cap pull-down RT-PCR	GCGACACAATAAGTTTTCATT
RT1103	pri-mir-1177	Semi-qRT-PCR<br>5'RACE (GS)-RT<br>Cap pull-down RT-PCR	GTAACCATAATGTAGTCATCAA
F1105	pri-mir-1177	Semi-qRT-PCR<br>Cap pull-down RT-PCR	GGAATGGTTACAAATCTTATAA
R681	pri-mir-1177	Semi-qRT-PCR<br>5'RACE (GS)<br>Cap pull-down RT-PCR	CCAGTTATTTTTCCTGGATAACA
F1025	upstream of pri-mir-1176	PCR verification of TSS	CATCGTTGTGACAAAACATTACC
ced37	3'DNA adaptor	3' RACE (LD)-RT	AGATCGGAAGAGCACACGTCT (Spacer C3)
ced41	Reverse primer	3' RACE (LD)-RT/PCR	AGACGTGTGCTCTTCCGATCT

F1023	pri-mir-1176	3'RACE	TCCCTCTGTCTCTTGATGATTCAG
F1022	pri-mir-1176	3'RACE (LD)	GCCCACCTGATACGACTGGGAAGTT
GeneRacer-Oligo dT(18)	(polyA)-RNA	3'RACE (oligo dT)-RT	GCTGTCAACGATACGCTACGTAACGGCATGACAGTG(T)18
GeneRacer 3' primer	Reverse primer	3'RACE (oligo dT)	GCTGTCAACGATACGCTACGTAACG
F1050	pri-mir-1176	3'-RACE (oligo dT)	GCCTTCCTTGACAAAAATTGCCC
RT1069	DSG and readthrough RNA	RT-PCR<br>5'RACE (GS)-RT	CTCCATAAGTGTCACCATTCTTTGC
F1066	pri-mir-1177	RT-PCR	CCAGTTAGGGTTTAATGGTTCTATC
R1068	pri-mir-1177	RT-PCR	GGGCTCAACGGTTCTTTTCCAGT
F1071	DSG	RT-PCR	GTGGTAATGACGTTGTTGGTTTATC
R1070	DSG	RT-PCR<br>5'RACE (GS)	CAACCAAACTATCCCCTACATAATC
F801	gpdA	Cap pull-down RT-PCR	GGTTGTCCCAATTGGTATTAATGG
R802	gpdA	Cap pull-down RT-PCR	CCGTGGGTTGAATCATATTTGAAC
RT975	gpdA	Cap pull-down RT-PCR	ATACCAGCATGAGCATCGAA
F1218	pri-mir-7097	RT-PCR	AATGGTCATTTAACTATTTACAAT
F1217	pri-mir-7097	RT-PCR	GTGATTGTTTTCACTAACCCACCC
R1221	pri-mir-7097	RT-PCR<br>5'RACE (oligo dT)	TCGTCTCTACTTCTGCCAAAATC

Supplementary Table S3. Input data for upstream motif analyses. The pri-miRNAs of intergenic miRNAs were predicted based on read coverage from poly(A) RNA-seq data. The genomic sequence upstream of the predicted start of each pri-miRNA were manually searched for a thymidine-rich region ending with a guanine residue.

miRNA	Predicted pri-miRNA location	Orientation	Motif	Motif location	Distance (nt) between motif and first poly(A) read
mir-1178	DDB0232429:1648949-1649613	-	TTTTTATTTTATTTTATTTAATTTTTG	DDB0232429:1649651-1649677	38
mir-1181	DDB0232428:2716790-2717868	-	TTATTTTTTTTTTTTACTGTTAATTTG	DDB0232428:2717988-2718014	120
mir-1183	DDB0232431:735430-736161	-	AATTAAATTTTGTTTATTTTTTTTTTG	DDB0232431:736183-736209	22
mir-1185	DDB0232433:957768-958754	-	ATTTTTTTTTATTTTATTTTCATTATG	DDB0232433:958753-958779	1
mir-1176	DDB0232432:171869-172380	+	AAATATTTTTTTTTTTTTTTTTTTTTG	DDB0232432:171835-171860	9
mir-1177	DDB0232432:593770-594156	+	TTTTTTTTTTTTTTTTTTTTTTTTTTG	DDB0232432:593814-593840	70
mir-7095	DDB0232429:2889969-2890628	-	TTTTGTTTGAAATTTGGTAGTTTTTTG	DDB0232429:2890769-2890795	141
mir-7097	DDB0232430:3738909-3739400	-	TTAATTTTTTTTTTTTTTTTTTTTTTG	DDB0232430:3739388-3739414	12
mir-7098	DDB0232429:886400-886823	+	TTTCTCTCTATTTTTTTTTTTTTTTTG	DDB0232429:886374-886400	0
mir-7103	DDB0232428:267885-268729	+	TAATTTATTTTTATATTTTTTTATATG	DDB0232428:267833-267859	26
mir-7104	DDB0232429:2338372-2338767	+	TTTTTTTTTTTTTTTGTTTTTTTTTTG	DDB0232429:2338331-2338346	26
mir-7105	DDB0232429:4787074-4789074	-	TTTTTTTTTTTTTTTTTTTTTTTTTTG	DDB0232429:4789143-4789167	69
miRNA_can_D1	DDB0232431:1706186-1706650	+	TTTAATTTGTTTTATATTAATTCACTG	DDB0232431:1706041-1706067	119
miRNA_can_D2	DDB0232432:4871472-4872110	+	TTTTTTTTTATTTTTAAAATTTCGTTG	DDB0232432:4871397-4871423	49
